# Neutrophils to the ROScue: Mechanisms of NADPH Oxidase Activation and Bacterial Resistance

**DOI:** 10.3389/fcimb.2017.00373

**Published:** 2017-08-25

**Authors:** Giang T. Nguyen, Erin R. Green, Joan Mecsas

**Affiliations:** ^1^Graduate Program in Immunology, Sackler School of Graduate Biomedical Sciences, Tufts University Boston, MA, United States; ^2^Department of Molecular Biology and Microbiology, Tufts University School of Medicine Boston, MA, United States

**Keywords:** reactive oxygen species, neutrophils, NADPH oxidase, G protein coupled receptors, Fc receptors, integrin receptors, type 3 secreted effectors, CGD

## Abstract

Reactive oxygen species (ROS) generated by NADPH oxidase play an important role in antimicrobial host defense and inflammation. Their deficiency in humans results in recurrent and severe bacterial infections, while their unregulated release leads to pathology from excessive inflammation. The release of high concentrations of ROS aids in clearance of invading bacteria. Localization of ROS release to phagosomes containing pathogens limits tissue damage. Host immune cells, like neutrophils, also known as PMNs, will release large amounts of ROS at the site of infection following the activation of surface receptors. The binding of ligands to G-protein-coupled receptors (GPCRs), toll-like receptors, and cytokine receptors can prime PMNs for a more robust response if additional signals are encountered. Meanwhile, activation of Fc and integrin directly induces high levels of ROS production. Additionally, GPCRs that bind to the bacterial-peptide analog fMLP, a neutrophil chemoattractant, can both prime cells and trigger low levels of ROS production. Engagement of these receptors initiates intracellular signaling pathways, resulting in activation of downstream effector proteins, assembly of the NADPH oxidase complex, and ultimately, the production of ROS by this complex. Within PMNs, ROS released by the NADPH oxidase complex can activate granular proteases and induce the formation of neutrophil extracellular traps (NETs). Additionally, ROS can cross the membranes of bacterial pathogens and damage their nucleic acids, proteins, and cell membranes. Consequently, in order to establish infections, bacterial pathogens employ various strategies to prevent restriction by PMN-derived ROS or downstream consequences of ROS production. Some pathogens are able to directly prevent the oxidative burst of phagocytes using secreted effector proteins or toxins that interfere with translocation of the NADPH oxidase complex or signaling pathways needed for its activation. Nonetheless, these pathogens often rely on repair and detoxifying proteins in addition to these secreted effectors and toxins in order to resist mammalian sources of ROS. This suggests that pathogens have both intrinsic and extrinsic mechanisms to avoid restriction by PMN-derived ROS. Here, we review mechanisms of oxidative burst in PMNs in response to bacterial infections, as well as the mechanisms by which bacterial pathogens thwart restriction by ROS to survive under conditions of oxidative stress.

## Introduction

Reactive oxygen species (ROS) production, i.e., oxidative burst, is a powerful antimicrobial weapon, and a major component of the innate immune defense against bacterial and fungal infections (Dupre-Crochet et al., 2013; Mocsai, 2013; Paiva and Bozza, 2014; Kruger et al., 2015; Van Acker and Coenye, 2017). Defects in ROS production allow bacteria to survive and repeatedly colonize various tissue sites as well as to cause septicemia (Baehner and Nathan, 1967; Holmes et al., 1967; Quie et al., 1967; van den Berg et al., 2009; Holland, 2013; Kulkarni et al., 2016; Wolach et al., 2017). While various cell types can produce ROS by different machineries to regulate and influence cellular processes (Trachootham et al., 2008; Bae et al., 2011; Ray et al., 2012; Nathan and Cunningham-Bussel, 2013; Navarro-Yepes et al., 2014; Reczek and Chandel, 2014; Schieber and Chandel, 2014; Gorlach et al., 2015), this review will focus on ROS generated by innate phagocytes, specifically by polymorphonuclear leukocytes (PMNs) via the multi-protein membrane-bound NADPH (Nicotinamide adenine dinucleotide phosphate-oxidase/Nox2) oxidase complex (Lambeth, 2004; Groemping and Rittinger, 2005; Bedard and Krause, 2007; Dupre-Crochet et al., 2013; Nunes et al., 2013; Paiva and Bozza, 2014; El-Benna et al., 2016). PMNs are the most abundant circulating white blood cells in humans, and produce inducible ROS via the NADPH oxidase complex (Lambeth, 2004; Mocsai, 2013). As both the first line of innate defense and effectors of adaptive immunity, PMNs play crucial roles in the immune defense against bacterial, fungal, and even viral infections (Mocsai, 2013; Kruger et al., 2015).

**Figure 1:**
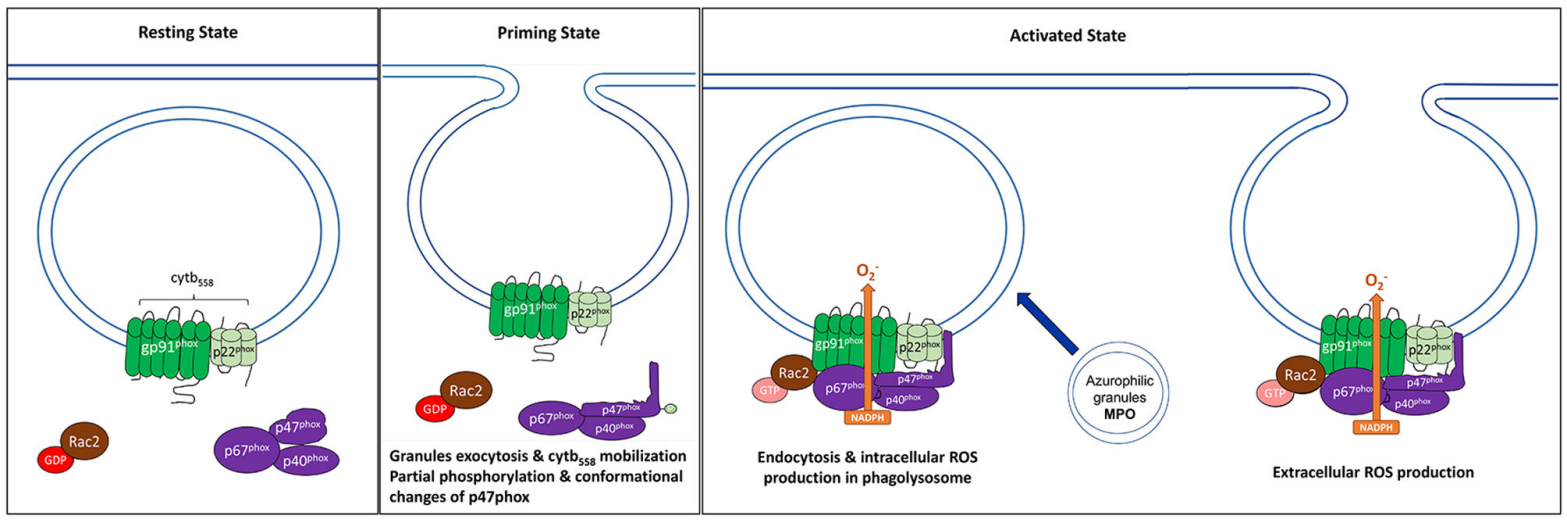
Components of the NADPH oxidase at resting and activated state. NADPH oxidase, also commonly referred to as the phagocyte oxidase (phox) complex, is a multi-protein electron transfer system that is made up of five components and Rac2. The catalytic core, also known as flavocytochrome b558 (cytb_558_), is a heterotrimeric dimer made up of two transmembrane proteins, gp91^phox^ and gp22^phox^. **(Left)** At resting state, cytb_558_ resides at the membranes of phagosomes, secretory vesicles, specific granules, and the plasma membrane and catalyzes the transfer of electrons from NADPH to molecular oxygen generating superoxide anions (${\text{O}}_{2}^{-}$) as by-products. Regulatory subunits, p40^phox^, p47^phox^, and p67^phox^, reside in the cytosol of resting cells. **(Center)** Priming induces several changes such as translocation of cytb_558_ to plasma membrane via granule exocytosis, partial phosphorylation of p47^phox^ leading to conformational changes. **(Right)** When PMNs are activated, the regulatory cytosolic complex translocates to the membrane and interacts with cytb_558_; this is required for NADPH activation. Another factor that regulates the recruitment of regulatory complex to the membranes and the overall activation of NADPH oxidase is small GTPase protein, Rac2. Activated GTP-bound Rac2 binds directly to gp91^phox^ and p67^phox^, and is also required for ROS production. For intracellular ROS production in the phagolysosome, this occurs after endocytosis of the complex. Meanwhile, extracellular ROS occurs at the plasma membrane.

Studies characterizing genetic mutations of the structural components of the NADPH oxidase complex have generated deeper insights into the importance of ROS in the host response to infection (Nunes et al., 2013; Paiva and Bozza, 2014; El-Benna et al., 2016). ROS can be released extracellularly into the environment at the site of infection or intracellularly in the phagolysosome following phagocytosis of bacteria (Figure 1) (Robinson, 2008; Dupre-Crochet et al., 2013; Nathan and Cunningham-Bussel, 2013). Importantly, ROS can further augment the overall antimicrobial response of PMNs by activating the release of granules, inducing the generation of neutrophil extracellular traps (NETs), and stimulating the production of the pro-inflammatory cytokines such as tumor necrosis factor alpha (TNFα) and macrophage inflammatory protein 2 (MIP-2) (Brinkmann et al., 2010; Naik and Dixit, 2011; Sheshachalam et al., 2014). In fact, these downstream effects of ROS production may ultimately be responsible for much of the bactericidal activities of ROS rather than direct damage by ROS themselves (Miralda et al., 2017). While mammalian hosts have developed potent ROS-dependent killing mechanisms, bacteria have also evolved various strategies to resist the bactericidal effects of ROS, both by directly impeding the generation of ROS as well as detoxifying ROS before they damage bacterial components. Thus, during an infection, there is always a tug-of-war between the invading species and the host immune response.

**Figure 2:**
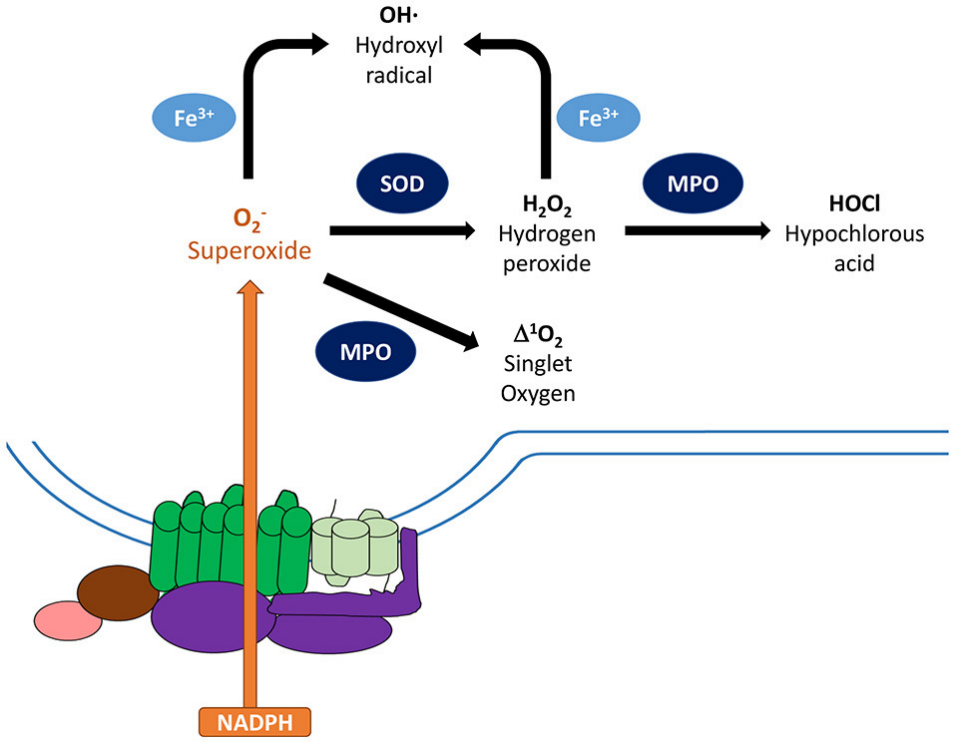
Metabolism of reactive oxygen species. Activated NADPH oxidase catalyzes the transfer of electrons from NADPH to molecular oxygen generating superoxide anions (${\text{O}}_{2}^{-}$) as the primary product. To minimize damage, cells are equipped with antioxidant scavenging enzymes, such as superoxide dismutase (SOD), which dismutates ${\text{O}}_{2}^{-}$ to non-radical species hydrogen peroxide (H_2_O_2_), and catalase. SOD and glutathione peroxidase can further convert these species into water, which limit damages to the host. On the other hand, ${\text{O}}_{2}^{-}$ can be converted to other reactive oxygen species that can damage nucleic acids, proteins, and cell membranes. Granule-localized myeloperoxidase (MPO) can convert H_2_O_2_ to hypochlorous acid (HOCl), which can enhance clearance of invading pathogens. MPO can also directly convert ${\text{O}}_{2}^{-}$ into singlet oxygen (^1^O_2_^*^). In addition, ferric iron can convert ${\text{O}}_{2}^{-}$ and H_2_O_2_ into hydroxyl radical (OH). Components of the NADPH oxidase: gp91^phox^ (green), gp22phox (light green), regulatory factors (purple).

A brisk immune response frequently clears a pathogen, but can cause significant local and, in some cases, long-term damage. Extensive damage can contribute to tissue injury, neurodegeneration, carcinogenesis, and aging (Trachootham et al., 2008; O'Neill et al., 2015). Additionally, when uncontrolled, the antimicrobial activities of PMNs can provoke severe inflammatory and autoimmune diseases, including systemic lupus erythematosus, rheumatoid arthritis, and type I diabetes (Kolaczkowska and Kubes, 2013). Thus, a well-regulated response is important for the health status of an individual. Consequently, the activation of oxidative burst must be tightly regulated and checkpoints exist to restrict the times and locations that are appropriate for cellular functions (Nathan and Cunningham-Bussel, 2013). Understanding how PMNs are activated and how they can become dysregulated should help to develop strategies to maintain the crucial balance between their beneficial and detrimental effects. Progress has been made in identifying proteins involved in relaying signals from receptors to the NADPH oxidase complex in PMNs. In addition, much work has been done to understand how the NADPH oxidase complex itself is assembled and regulated which leads to oxidative burst in these cells. Here, we will summarize the current molecular understanding of this priming and activation of the NADPH oxidase and provide a more thorough discussion of the activation of receptors that ultimately lead to the activation of the complex and the tug-of-war between ROS production between PMNs and bacterial pathogens.

## CGD: genetic diseases of NADPH-oxidase

Chronic Granulomatous Disease (CGD) is a rare inherited immunodeficiency syndrome that affects one out of every 200,000–250,000 live human births (Holland, 2013; O'Neill et al., 2015; Dinauer, 2016). CGD is caused by mutations in genes encoding components of the NADPH oxidase complex, leading to a defect in ROS production by phagocytes (Table 1) (Baehner and Nathan, 1967; Holmes et al., 1967; Quie et al., 1967; Segal and Jones, 1978; O'Neill et al., 2015; Dinauer, 2016). Mutations that cause CGD are found in *CYBB* and *CYBA*, which encode the membrane-bound NADPH oxidase components Nox2/gp91^phox^ and p22^phox^, and *NCF1, NCF2*, and *NCF4*, which encode the cytosolic regulatory factors p47^phox^, p67^phox^, and p40^phox^, respectively (Figure 1) (O'Neill et al., 2015). As a result of the failure of phagocytes to mount a respiratory burst, the majority of CGD patients are susceptible to recurrent and life-threatening bacterial and fungal infections early in childhood, due to ineffective killing and containment of the pathogens (van den Berg et al., 2009; Holland, 2013; O'Neill et al., 2015). Common infectious syndromes resulting from CGD include pneumonia and lung abscesses, and these patients are commonly infected by gram-positive bacteria (*Staphylococcus aureus*), gram-negative bacteria (*Salmonella*), and fungi (*Aspergillus, Candida albicans*) (Holland, 2013). Approximately 65% of CGD patients have an X-linked mutation in *CYBB* gene, which is the major genetic form of CGD (van den Berg et al., 2009; Holland, 2013; Kulkarni et al., 2016; Wolach et al., 2017). Meanwhile, autosomal mutations in *CYBA, NCF1, NCF2*, and *NCF4* cause autosomal recessive CGD. About 25% of patients carry mutations in their *NCF1* gene, while mutations in *CYBA, NCF2*, and *NCF4* are more rare.

**Table 1:** The genes and proteins causing chronic granulomatous disease.

**Location at resting state**	**Gene name**	**NADPH oxidase protein component**	**Inheritance**
Membrane	CYBB	gp91^phox^/NOX2	X-linked
	CYBA	p22^phox^	Autosomal
Cytoplasmic	NCF1	p47^phox^	Autosomal
	NCF2	p67^phox^	Autosomal
	NCF4	p40^phox^	Autosomal

Although those suffering from CGD exhibit a wide range of clinical symptoms, ranging from a relatively mild presentation late in life to fatal septicemia in infancy, X-linked *CYBB* CGD generally causes more severe infections and earlier deaths than autosomal recessive CGD (Holland, 2013; Dinauer, 2016). This is due, in part, to the fact that *CYBB* encodes the cytochrome subunit gp91^phox^. Different genetic mutations in *CYBB* can modulate the level of superoxide that PMNs are able to generate, thus dictating how susceptible the individual is to infections (Royer-Pokora et al., 1986; Rae et al., 1998). For example, a mutation in the catalytic domain of Nox2 or in the domain responsible for interacting with the other NADPH subunits leads to a total loss of oxidative burst, whereas some mutations in the dehydrogenase domain have no effect on ROS production by phagocytes (Holland, 2013; O'Neill et al., 2015; Dinauer, 2016). In addition to exhibiting increased susceptibility to infections, some CGD patients also develop large diffuse granulomas that can cause obstructions or painful symptoms in the affected areas, such as the esophagus and stomach. Some CGD patients also suffer from dysfunctional disorders due to extensive fibrosis in all areas of the body (van den Berg et al., 2009; Kulkarni et al., 2016; Wolach et al., 2017), which has been correlated with chronic inflammation associated with the disease. Despite decades of research, it remains challenging to determine the proper course of treatment for a particular CGD patient, as symptoms can develop over the patient's lifetime.

## General structure and components of the NADPH oxidase

The NADPH components are dormant in resting cells and become activated in response to pro-inflammatory mediators, the presence of microbes, phagocytosis, and/or the activation of pattern recognition receptors (PRRs). The phagocyte oxidase (phox) complex includes five subunits: gp91^phox^, p22^phox^, p40^phox^, p47^phox^, and p67^phox^ (El-Benna et al., 2016). In their resting state, gp91^phox^ and p22^phox^ form a heterodimeric subunit, flavocytochrome b558 (cytb_558_), which constitutes the catalytic core of the NADPH oxidase and resides at cellular membranes, including the membranes of phagosomes, secretory vesicles, specific granules, and the plasma membrane (Groemping and Rittinger, 2005; Bedard and Krause, 2007; Nathan and Cunningham-Bussel, 2013; Nunes et al., 2013; El-Benna et al., 2016). gp91^phox^ is the electron transferase of NADPH oxidase. Its cytosolic domain accepts electrons from NADPH, and transfers them across the membrane to O_2_ to generate superoxide (${\text{O}}_{2}^{-}$) (Figure 2) (Cross and Segal, 2004; Groemping and Rittinger, 2005; Nunes et al., 2013; Panday et al., 2015; El-Benna et al., 2016). p22^phox^ acts as a docking site for the regulatory trimeric complex via its interaction with p47^phox^ (Lewis et al., 2010). The regulatory complex, comprised of p40^phox^, p47^phox^, and p67^phox^, resides as a complex in the cytosol of dormant cells (Figure 1) (Nunes et al., 2013; El-Benna et al., 2016). The separation of the oxidase complex components into two groups and their distribution between distinct subcellular compartments of the cell prevents spontaneous activation and potential damage in the resting host cell. Furthermore, this separation provides multiple points of regulation of the ROS production and will be discussed in detail in section “Assembly and Activation of NADPH Oxidase.” Upon activation, the regulatory complex interacts with cytb_558_ to promote electron transfer from NADPH to Flavin adenine dinucleotide (FAD) (Cross and Segal, 2004; Nunes et al., 2013). In addition to these oxidase-specific subunits, the small GTPase protein Rac2 is an essential subunit and is sequestered in the cytosol as Rac-GDP in resting cells (Kim and Dinauer, 2001; Miyano and Sumimoto, 2012).

Further levels of regulation, such as those provided by Ca^2+^ signaling and phosphorylation cascades, occur after priming and/or activation of receptors and control the recruitment of the regulatory components to membranes to activate the NADPH complex (Kim and Dinauer, 2001; Bokoch and Zhao, 2006; El-Benna et al., 2009; Raad et al., 2009; Gorlach et al., 2015). In brief, upon stimulation, assembly of the NADPH oxidase is initiated by two simultaneous events: the activation of Rac2 via the exchange of guanosine diphosphate (GDP) for guanosine triphosphate (GTP) and the phosphorylation of p47^phox^ at multiple serine sites (Nunes et al., 2013; El-Benna et al., 2016). Upon activation, Rac2 and the phosphorylated p47^phox^/p40^phox^/p67^phox^ complex translocate simultaneously, but independently of each other, to the membrane to interact with cytb_558_, forming the NADPH oxidase complex (Heyworth et al., 1994; Kim and Dinauer, 2006). Once formed, the NADPH oxidase complex facilitates the transfer of electrons from the cytosol to oxygen, ultimately generating superoxide anions.

## Receptor-mediated signaling pathways regulating NADPH oxidase activation in PMNs

**Figure 3:**
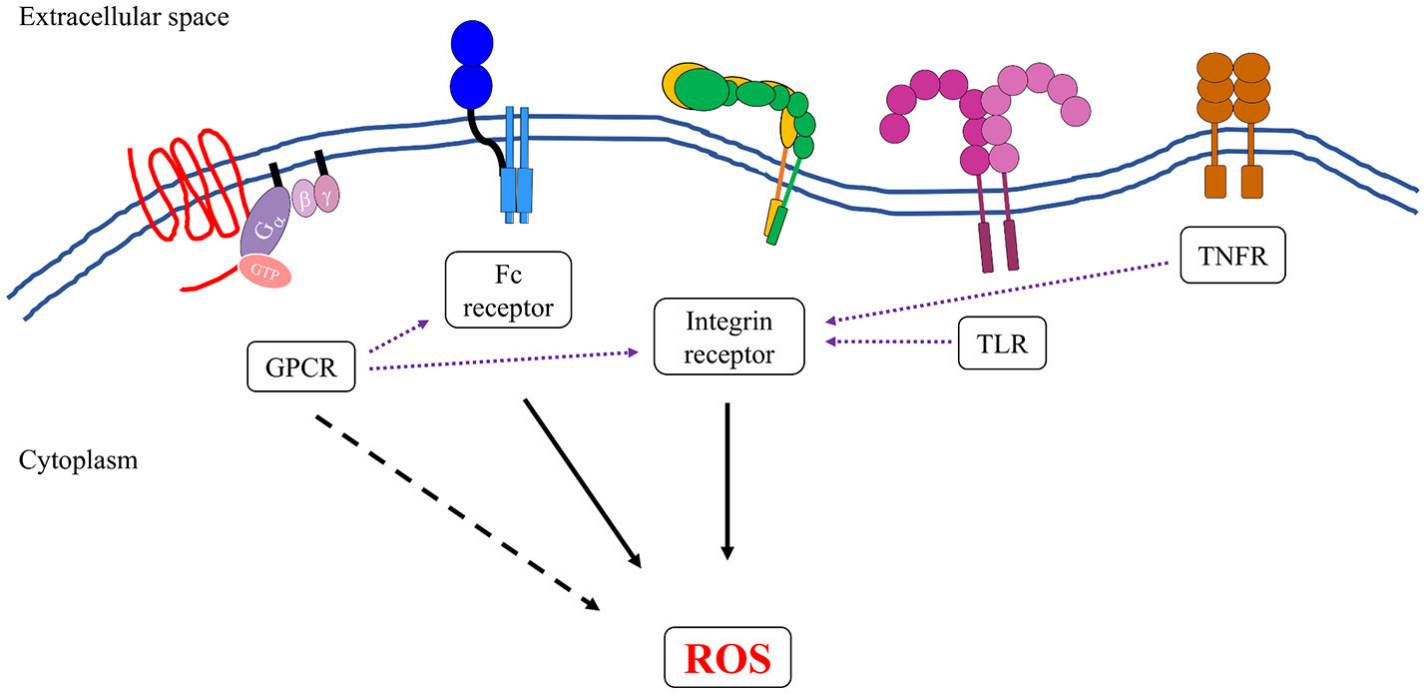
Neutrophils express several groups of receptors that can induce the formation and generation of reactive oxygen species. Activation of integrin and Fc receptors leads to complex intracellular signal transduction pathways that can robustly activate the NADPH oxidase complex (solid black arrows). Some members of G-protein-coupled receptors (GPCRs) family, specifically formyl receptors, can directly activate NADPH oxidase, although to a lesser extent than to what has been observed in integrin and Fc receptors (dotted black arrow). Ligand binding to TLRs, TNFRs, and some members of GPCRs can transform the neutrophils into an “primed” state, whereby the NADPH oxidase is more susceptible to activation by a secondary stimulus (purple dotted arrows). This is presumably another level of regulation to ensure that reactive oxygen species are produced at the right time and place that is only during an active infection.

At the site of infection, PMNs express a large number of cell surface receptors that recognize the presence of pathogens or other markers of the inflammatory environment (Futosi et al., 2013). Activation of these receptors in PMNs triggers a variety of intracellular signaling pathways that support an efficient antimicrobial response, including ROS production (Figure 3), and promote an inflammatory environment. In this discussion, the term “activation” refers to a ligand-dependent response leading to detectable superoxide production from one stimulus (Figure 3). By contrast, priming refers to the transformation of PMNs following exposure to a ligand that does not itself induce superoxide production, but does render the PMNs more amenable to robust activation of NADPH oxidase upon binding to a second ligand (El-Benna et al., 2008, 2016). This intermediary activation or primed state occurs following preparation of the cell and the NADPH oxidase by the first stimulus, resulting in stronger activation by the second stimulus (El-Benna et al., 2016; Miralda et al., 2017). Both priming and activation are blocked by treatment with genistein, a tyrosine kinase inhibitor, suggesting an important role for tyrosine kinase signaling pathway in activating NADPH oxidase (McLeish et al., 1998; Dang et al., 2006). Here, we will first introduce the receptors that have been shown to prime cells for activation. For additional reviews on priming effects on PMNs (see the following reviews El-Benna et al., 2016; Miralda et al., 2017). Next, we will discuss how the proximal signals from integrin and Fc receptors that directly activate the NADPH oxidase complex (Berton et al., 1992; Dewas et al., 2000; Mocsai et al., 2002, 2006; Newbrough et al., 2003; Clemens et al., 2004; Gakidis et al., 2004; Kahn and Koretzky, 2006; Fumagalli et al., 2007; Jakus et al., 2008, 2009; Lawson et al., 2011; Futosi et al., 2013). In section “Assembly and Activation of NADPH Oxidase,” we will discuss some of the molecular changes to the regulatory subunits that occur upon priming and activation.

### Priming for neutrophil oxidative burst

Signals from G protein coupled receptors (GPCRs), cytokine receptors such as Tumor Necrosis Factor receptors (TNFRs), and Toll-like Receptors (TLRs) can prime the cell for a more robust activation of the NADPH oxidase complex (El-Benna et al., 2008, 2016). These signals induce a variety of changes to the cell, including the partial phosphorylation of p47^phox^, conformational changes in the p47^phox^/p40^phox^/p67^phox^ complex, and the translocation of cytb_558_ from intracellular granules to the plasma membrane (Hallett and Lloyds, 1995; El-Benna et al., 2016). By contrast, some integrin receptors, Fc receptors, and the GPCR recognizing N-Formylmethionine-leucyl-phenylalanine (fMLP) can activate the NADPH oxidase complex directly.

#### GPCRs

**Figure 4:**
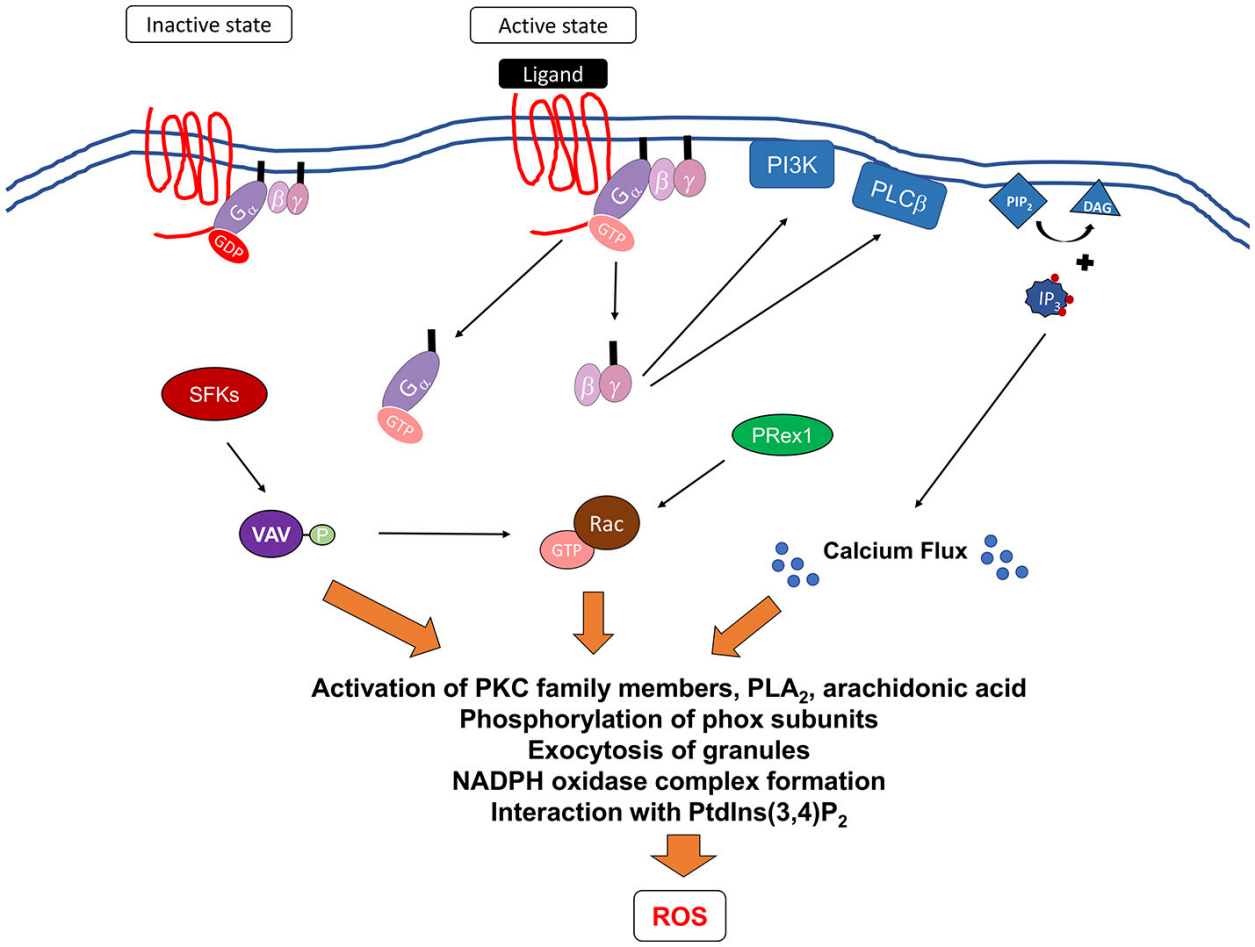
Signaling pathways mediating formyl receptor (GPCR)-induced NADPH oxidase activation. Ligation of G-protein-coupled receptors leads to changes in the receptor conformation resulting in the exchange of GDP for GTP bound to the G protein. This leads to the dissociation of the G proteins subunits, G_α_ and G_βγ_ from the membrane to activate downstream effectors. It is currently unclear how G_α_ contributes to the activation of NADPH oxidase. G_βγ_ can activate PI3K, which can act to mediate PRex1-dependent Rac2 activation, and PLCβ, which leads to the breakdown of membrane phospholipid, PIP2, into DAG and IP_3_. DAG induces calcium flux, while IP_3_ can act on further downstream proteins. In addition, Src family kinases (SFKs) have been shown to be important and may activate Vav proteins leading to the activation of p38 MAPK and potentially Rac2. Activation of these proximal signaling molecules lead to exocytosis of granules, activation of various PKC family members, phospholipase A_2_ (PLA_2_), and release of arachidonic acid, a lipid messenger. All of these secondary messengers are required for phosphorylation of phox subunits, formation of NADPH oxidase, and interaction with phosphatidylinositol 3,4-biphosphate (PtdIns(3,4)*P*_2_).

In healthy individuals, resting PMNs freely circulate in the body (Kolaczkowska and Kubes, 2013). Upon infection, resident epithelial cells, macrophages, and the complement system release pro-inflammatory mediators that induce changes in the vascular epithelium, which in turn signals to the circulating PMNs to roll, adhere, and cross the endothelial barrier (Kolaczkowska and Kubes, 2013). PMNs are then directed to the infection site through GPCR recognition of a gradient of locally produced chemoattractants and inflammatory agents, including interleukin-8 (IL-8), platelet activating factor (PAF), leukotriene B4 (LT4), complement factor C5a, and the bacterial-peptide analog, fMLP, a neutrophil chemoattractant (Migeotte et al., 2006; Rabiet et al., 2007; Kolaczkowska and Kubes, 2013; Bloes et al., 2015; El-Benna et al., 2016). GPCRs are characterized by their seven transmembrane topology and their linkage to heterotrimeric GTPase (G) proteins on their cytosol, and represent the largest class of membrane proteins in the human genome (Figure 4) (Fredriksson et al., 2003).

The binding of agonists to the extracellular domain of a GPCR stabilizes the active conformation of the receptor (Katritch et al., 2013; Ghosh et al., 2015), which in turn triggers the exchange of GDP for GTP by the G proteins. This leads to the dissociation of the G protein subunits, G_α_ and G_βγ_, from the receptor's cytoplasmic tail (Selvatici et al., 2006; Futosi et al., 2013; Katritch et al., 2013; Ghosh et al., 2015), and the subsequent activation of downstream pathways. Experiments performed with human PMNs have shown that the binding of IL-8 to its receptor induces the activation of phospholipase A_2_ (PLA_2_), calcium release, and upregulation of the surface expression of N-formyl peptide receptors (Daniels et al., 1992; Wozniak et al., 1993; El-Benna et al., 2016). IL-8 and PAF can also initiate the phosphorylation and translocation of p47^phox^ and p67^phox^ to the plasma membrane (Brown et al., 2004; Guichard et al., 2005; El-Benna et al., 2016).

#### Cytokine receptors

Following their migration and extravasation from the bloodstream into the tissue, PMNs are rapidly activated by proinflammatory cytokines, which are detected by surface cytokine receptors (Kato and Kitagawa, 2006; Futosi et al., 2013). For detailed reviews on how cytokine receptors prime ROS production, see Kato and Kitagawa (2006); El-Benna et al. (2016). TNF-α can prime cells for oxidative burst by inducing calcium flux and p38 MAPK-dependent mobilization of cytb_558_ to the plasma membrane (Yuo et al., 1989; Ward et al., 2000; Brown et al., 2004). Priming of PMNs by granulocyte macrophage colony stimulating factor (GM-CSF) or TNF-α leads to the phosphorylation of p47^phox^ Ser345 (pSer345) (Dang et al., 2006). Phosphorylation of this site enhances additional phosphorylation of other sites and promoting the translocation and docking of the cytosolic complex to the membrane (Dang et al., 2006; El-Benna et al., 2016). GM-CSF primes human PMNs by inducing phosphorylation of Ser345 (pSer345) via ERK1/2, while TNF-α-primes by p38MAPK-mediated pSer345 (Dang et al., 2006; El-Benna et al., 2016). Phosphorylation of Ser345 is thought to potentiate the phosphorylation of other residues by activation of Pin1, a proline isomerase (Boussetta et al., 2010; Makni-Maalej et al., 2012). This leads to enhanced conformational changes in Pin1 and in turn changes in p47^phox^ conformation, which reveals additional phosphorylation sites on p47^phox^ for phosphorylation (Dang et al., 2006; Kato and Kitagawa, 2006; El-Benna et al., 2016). Additionally, priming of PMNs by TNF-α induces p38 mitogen-activated protein kinase (MAPK)-dependent phosphorylation of p67^phox^ (Brown et al., 2004), which causes conformational changes in p67^phox^, allowing it to interact with gp91^phox^ (Dang et al., 2006; El-Benna et al., 2016). Furthermore, work in human PMNs has shown that prolyl isomerase Pin1 can subsequently bind to pSer345 following TNF-α stimulation, which exposes additional amino acids for phosphorylation by protein kinase C (PKC) (Boussetta et al., 2010).

#### Toll-like receptors

PMNs express a broad range of PRRs that are involved in the direct recognition of invading pathogens (Kawasaki and Kawai, 2014). Members of TLRs are present on the cell surface and intracellular endocytic compartments of PMNs (Kawasaki and Kawai, 2014). TLRs recognize a variety of microbial structures, including lipopolysaccharide (LPS) by TLR4, flagellin by TLR5, and peptidoglycan by TLR2. Specifically, treatment with LPS has been shown to partially phosphorylate and induce the translocation of p47^phox^ (Ward et al., 2000; Brown et al., 2004). Activation of TLR4 by LPS can also increase the expression of gp91^phox^ at the plasma membrane via p38 (DeLeo et al., 1998). Likewise activation of TLR7 induces phosphorylation of gp41phox and activation of Pin1 (Makni-Maalej et al., 2015; El-Benna et al., 2016).

### Direct activation of oxidative burst

#### GPCRs recognizing fMLP

Unlike other GPCRs, formyl receptors, which recognize fMLP, have dual effects on PMNs, as they can either prime the cells or activate NADPH oxidase directly (Dang et al., 2001; Migeotte et al., 2006; Selvatici et al., 2006; Fumagalli et al., 2007; Rabiet et al., 2007; Lawson et al., 2011). Here, we will discuss the known signaling proteins mediating fMLP-induced oxidative burst. The binding of fMLP to its receptors leads to the dissociation of the G protein subunits. These subunits then activate other downstream signaling proteins to generate secondary messengers such as cAMP, inositol phosphates, and Ca^2+^, resulting in a variety of cellular responses, including ROS production (Figure 4) (Ali et al., 1998). In particular, the G_βγ_ subunits activate both phospholipase beta (PLCβ) and class I phosphoinositide 3 kinase (PI3K)-dependent signaling cascades (Camps et al., 1992; Stephens et al., 1994). PLCβ enzymes are responsible for the generation of inositol trisphosphate (IP_3_), which in turn leads to the release of intracellular Ca^2+^ stores (Li et al., 2000), a requirement for oxidative burst (Gorlach et al., 2015). Interestingly, genetic deficiency in two PLCβ isoforms, PLCβ2 and PLCβ3, in PMNs leads to the abolishment of fMLP-induced superoxide production. However, the loss of just PLCβ2 is sufficient to reduce ROS production to level slightly above resting cells (Li et al., 2000), suggesting that PLCβ2 is the primary mediator of superoxide production.

PI3K catalyzes the synthesis of the second messenger phosphatidylinositol 3,4,5-trisphosphate (PIP_3_) (Hawkins et al., 2010; Houslay, 2016). In particular, it has been demonstrated that PI3Kγ, and more recently, PI3K_β_ isoforms, are required for superoxide production (Hirsch et al., 2000; Li et al., 2000; Houslay, 2016). Intriguingly, there is evidence that class I PIK3s can activate Rac2 through regulation of one of its guanine exchange factor (GEF), PRex1 (Kim and Dinauer, 2001; Dong et al., 2005; Lawson et al., 2011). However, there are many other GEFs, including CDM family members DOCK2 and DOCK5 (Watanabe et al., 2014), that also exert effects on downstream signaling molecules and the components of NADPH oxidase.

Src family kinases (SFKs), specifically Hck and Fgr, are involved in signal transduction after GPCR engagement and are important for fMLP-induced superoxide production. Specifically, genetic loss of Hck and Fgr leads to a reduction in the activation of p38 MAPK, JNK kinases, and the Vav1-Rac2-PAK pathways after fMLP stimulation (Fumagalli et al., 2007, 2013). Supporting these findings, human PMNs treated with p38 MAPK inhibitors and PMNs isolated from mice deficient in Vav1 are defective for fMLP-induced superoxide production (Yan et al., 2002; Kim et al, 2003). During infection, Src kinase-, PLCβ-, and PI3K-mediated pathways may act in parallel or there may be crosstalk among these pathways.

Mice deficient in a guanine activating protein (GAP), GIT2, produce more ROS than wild-type mice when stimulated with fMLP or complement factor C5a (Mazaki et al., 2006); furthermore, the resulting superoxide burst was often misdirected away from the chemoattractant source, which could explain why these *GIT2*^−/−^ mice are immunodeficient (Mazaki et al., 2006). These findings suggest that signal transduction pathways downstream of GPCR activation contain negative feedback loops and may interact with the cytoskeletal system in order to direct ROS toward the correct location in order to limit damage to the host.

#### Fc receptors

**Figure 5:**
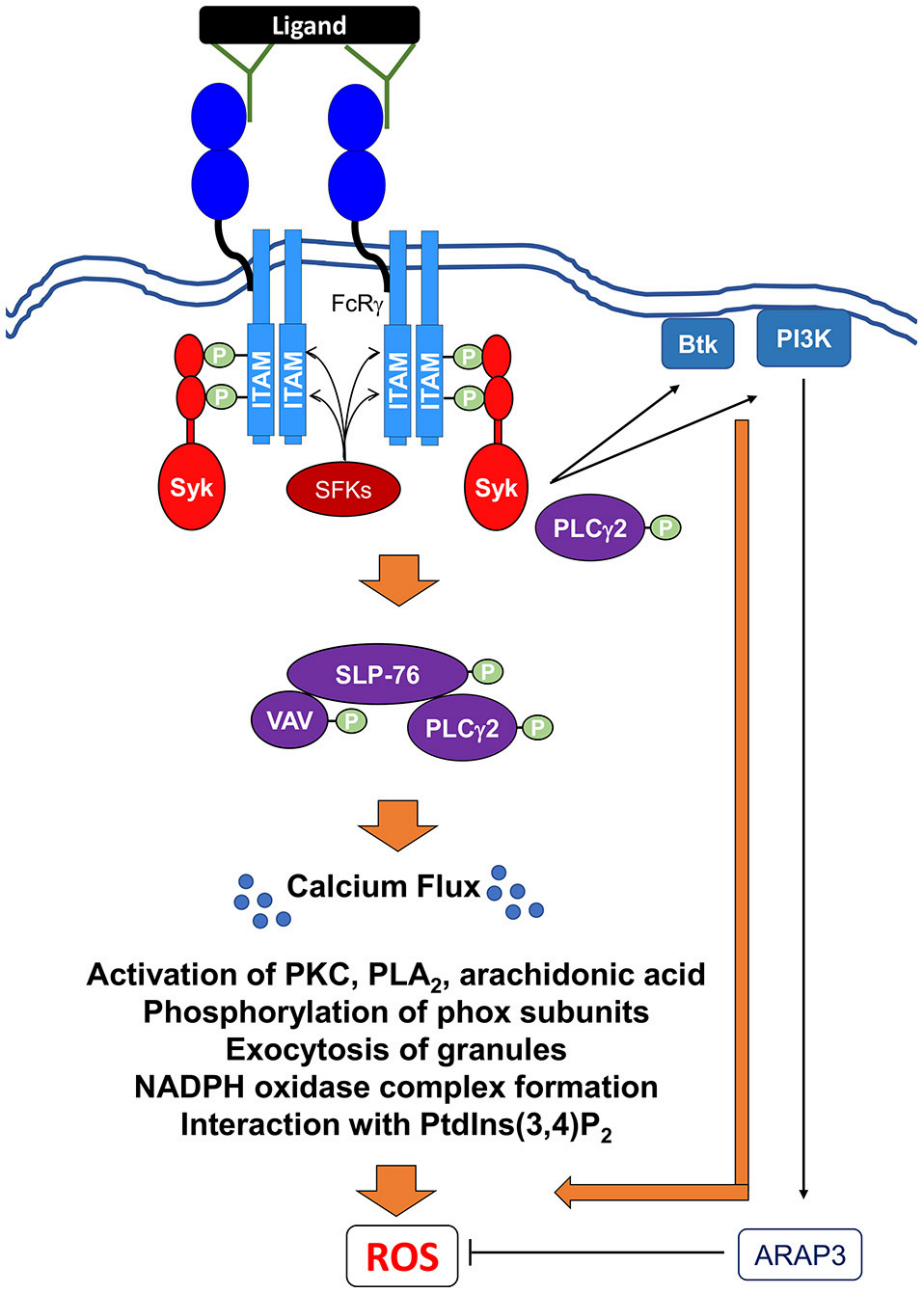
Signaling pathways mediating Fc receptor-induced NADPH oxidase activation via IgG immune complexes. Ligation and crosslinking of Fc receptors leads to the phosphorylation of the ITAMs by Src family kinases (SFKs) resulting in the recruitment and the tyrosine phosphorylation of the Src homology domain of Syk. Activated Syk can then recruit and activate Btk (Bruton's tyrosine kinase), class I PI3K (phosphoinositide 3-kinase). A class I PI3K effector, ARAP3, has been shown to negatively regulate ROS production (Gambardella et al., 2013). Syk also induces the formation and activation of the SLP76 signaling complex, which includes SLP76, Vav, and PLCγ2. Activation of this complex leads to further downstream effectors resulting in the release of intracellular calcium stores (Ca^2+^ flux), which is critical for ROS production. In addition, PLCγ2 can potentially interact directly with Syk to perpetuate the signal for ROS production. Activation of these proximal signaling molecules lead to exocytosis of granules, activation of various PKC family members, phospholipase A_2_ (PLA_2_), and release of arachidonic acid, a lipid messenger. All of these secondary messengers are required for phosphorylation of phox subunits, formation of NADPH oxidase, and interaction with phosphatidylinositol 3,4-biphosphate (PtdIns(3,4)*P*_2_).

PMNs express both high and low-affinity Fc receptors that are primarily involved in the recognition and phagocytosis of antibody-opsonized pathogens. However, Fc receptors can also participate in the induction of ROS production in these cells (Garcia-Garcia and Rosales, 2002). Specifically, low-affinity Fcγ receptors (FcγRs) are transmembrane proteins that bind to the Fc portion of IgG and signal through their ITAM (immunoreceptor tyrosine-based activation motif) domains (Bruhns, 2012). Humans express FcγRIIA, a single transmembrane receptor with an ITAM in its cytoplasmic tail, and FcγRIIIB, a GPI-anchored extracellular receptor (Jakus et al., 2008; Futosi et al., 2013). Functionally important single nucleotide polymorphisms have been described in these two Fc receptors (Huizinga et al., 1990a,b; Minchinton et al., 1995; Buxhofer-Ausch et al., 2014). The combination of FcγRIIA and FcγRIIIB isoforms expressed on PMNs influenced IgG immune complex (IgG IC)-mediated ROS production (van der Heijden et al., 2014). Mice express high levels of FcγRIII and FcγRIV, which are both multimeric receptors that non-covalently associate with FcRγ, an ITAM transmembrane adapter protein containing a short extracellular domain, a transmembrane segment, and a cytoplasmic tail (Murphy, 2012; Futosi et al., 2013). In mice, the receptors FcγRIII and FcγRIV, and the ITAM carrying protein, FcRγ are all required for IgG-mediated activation of superoxide production (Jakus et al., 2008). Human PMNs require signaling through FcγRIIA or FcγRIIIB to elicit ROS production by IgG IC (Jakus et al., 2008). PMNs activated by the binding of IgG to FcγRs elicit similar or higher levels of superoxide production than PMNs activated by β2 integrin (discussed below), indicating that Fc-mediated activation produces a very robust response. Opsonized bacteria are also phagocytosed by PMNs, which can enhance ROS production via the induction of receptors by degraded bacterial products. It has been also reported that complement receptor 3 (β2 integrin) and FcγRIII can cooperate to generate PMNs ROS production (Zhou and Brown, 1994); however, a more recent report has shown that blocking of FcγRIII by antibodies is sufficient to inhibit IgG ICs-mediated ROS production (Jakus et al., 2009). Ligand binding leads to the phosphorylation of ITAM sequences by SFK (Mocsai et al., 2011). While *Hck*^−/−^ *Fgr*^−/−^ neutrophils can produce ROS normally in response to IgG ICs-activation, *Hck*^−/−^ *Fgr*^−/−^ *Lyn*^−/−^ PMNs have defective ROS production in response to FcγR-mediated phagocytosis and IgG ICs (Lowell et al., 1996; Paul et al., 2008; Kovacs et al., 2014). Importantly, FcRγ recruits and activates Syk through the binding of the phosphotyrosine of its ITAM domain to SH2 domains of Syk (Figure 5) (Mocsai et al., 2011).

Syk transmits signals to a number of proteins including Btk, a Tec family kinase, and PI3K, whose activation is required for ROS production following FcR stimulation (Figure 5) (Kulkani et al., 2011; Fumagalli et al., 2013; Volmering et al., 2016). Upon FcR stimulation by IgG IC, Tec family kinases are translocated to the plasma membrane and phosphorylated in human PMNs in a PI3K- and SFKs-dependent mechanism (Fernandes et al., 2005). Specifically, *Btk*^−/−^ PMNs fail to produce superoxide production when plated on IgG-coated surfaces, indicating their importance downstream of Fcγ receptor activation (Volmering et al., 2016). As with GPCR stimulation, PMNs lacking PI3Kβ fail to produce ROS in response to IgG IC stimulation (Kulkani et al., 2011). Interestingly, PMNs expressing a kinase-deficient PI3Kβ can still undergo oxidative burst, but fail to produce ROS when the ATP-binding site of this protein is blocked by inhibitors (Kulkani et al., 2011). Combined, these observations suggest that the ability of PI3Kβ to initiate ROS production is independent of its kinase activity (Kulkani et al., 2011), but instead requires its ATP binding activity to stimulate phosphorylation of Akt and ERK downstream of FcγR activation. A known effector of PI3K, ARAP3, a GAP for small GTPases like RhoA and Arf6 has been shown to be a negative regulator of IgG IC-activated ROS production (Gambardella et al., 2013).

Activation of Syk is also critical for relaying signals to the adaptor protein SLP-76 and its effectors Vav and PLCγ2, all of which are critical for ROS production (Newbrough et al., 2003; Utomo et al., 2006). Notably, *SLP-76*^−/−^ PMNs produce lower levels of ROS following IgG IC activation, which suggests two possibilities (Newbrough et al., 2003): either another protein plays a partially redundant role in activating NADPH oxidase following FcγR activation, or FcγR stimulation could activate two independent pathways. In PMNs, the loss of the Vav GEF family member Vav3 abrogates ROS production (Utomo et al., 2006), as Vav is required for both the activation of Rac2 and the phosphorylation of p40^phox^ (Kim and Dinauer, 2001; Utomo et al., 2006). Additionally, *PLC*γ*2*^−/−^ PMNs fail to generate ROS in response to IgG IC stimulation (Jakus et al., 2009). The role of PLCγ2 is complex, as it appears to be activated via two different mechanisms, one SLP-76-dependent and the other SLP-76-independent, following FcγR activation (Jakus et al., 2009). It is likely that FcγR activation of ROS production also requires several of the proteins involved in ITAM-mediated signaling downstream of integrin activation (Love and Hayes, 2010). It is important to note that IC activation differs from Fc-receptor-mediated phagocytosis. For instance, further downstream, NADPH oxidase activation in response to FcR-mediated phagocytosis is dependent on the binding of p40^phox^ to PI(3)P, Rac2, and Rab27a (Forsberg et al., 2003; Anderson et al., 2010). In addition, Cdc42, a member of Rho GTPase family, PAK, and phospholipase D are activated downstream of FcR-phagocytosis-mediated ROS production (Lofgren et al., 1999; Forsberg et al., 2003).

#### Integrin receptors

Integrin receptors are large transmembrane glycoproteins that are made up of non-covalently associated α and β subunits and are present in virtually all mammalian cells (Harburger and Calderwood, 2009; Campbell and Humphries, 2011). While there are numerous α and β subunits, PMNs express the β_1_ (CD29), β_2_ (CD18), and β_3_ (CD61) integrins (coupled with various α subunits), which recognize fibronectin, fibrinogen, and collagen *in vitro*, respectively (Hynes, 1987). These receptors are important for the binding of PMNs to the extracellular matrix in order to facilitate adhesion and transmigration from the blood into infected tissues (Kolaczkowska and Kubes, 2013; Winograd-Katz et al., 2014). The loss of these receptors can lead to defects in leukocyte adhesion and migration, resulting in various forms of leukocyte adhesion deficiency (LAD), a genetic disorder characterized by severe bacterial infections in humans (Abram and Lowell, 2009; Kolaczkowska and Kubes, 2013; Winograd-Katz et al., 2014). Additionally, integrin receptors can bind to other cells or bacteria that bear appropriate receptor ligands, or to complement components. Activation of integrin receptors in PMNs leads to a signaling cascade that results in the phagocytosis of extracellular pathogens, as well as in superoxide burst (Williams and Solomkin, 1999).

**Figure 6:**
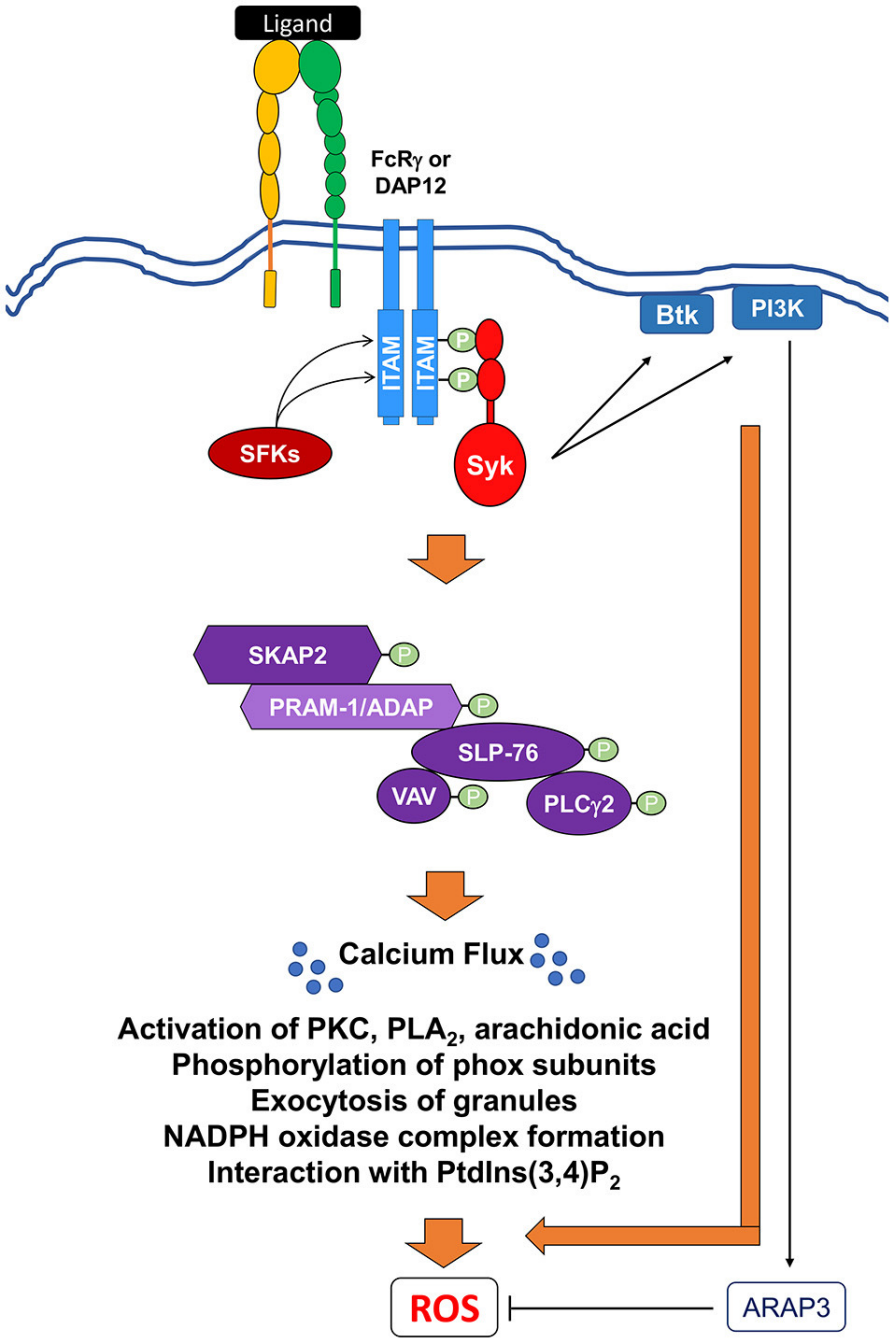
Signaling pathways mediating integrin-induced NADPH oxidase activation. Ligation and crosslinking of integrin receptors leads to the phosphorylation of the ITAM-containing proteins, DAP12 and FcRγ, by (SFKs), resulting in the recruitment and the tyrosine phosphorylation of the Src homology domain of Syk. Activated Syk can then act to recruit and activate Bruton's tyrosine kinase (Btk) and class I phosphoinositide 3-kinase (PI3K). A class I PI3K effector, ARAP3, has been shown to negatively regulate ROS production. Syk also induces the activation of SH2-domain-containing leukocyte protein of 76 kDa (SLP76) to form a multi-protein signaling complex. This SLP76 complex can then recruit and activate downstream effectors proteins like SKAP2, SLP76, the Vav GEF family, and PLCγ2. Activation of this complex leads to further downstream effectors resulting in the release of intracellular calcium stores (Ca^2+^ flux) and ultimate ROS production. Activation of these proximal signaling molecules lead to exocytosis of granules, activation of various PKC family members, phospholipase A_2_ (PLA_2_), and release of arachidonic acid, a lipid messenger. All of these secondary messengers are required for phosphorylation of phox subunits, formation of NADPH oxidase, and interaction with phosphatidylinositol 3,4-biphosphate (PtdIns(3,4)*P*_2_).

In an inflammatory environment, signaling by integrins in PMNs is regulated by two consecutive signaling pathways: “inside-out” and “outside-in” (Abram and Lowell, 2009; Campbell and Humphries, 2011). Intracellular inside-out signaling is induced by the binding of soluble ligands to receptors, including GPCRs, TNFRs, or TLRs, leading to the binding of cytoskeletal proteins talin and kindlin to the cytoplasmic domains of the integrin β subunit (Abram and Lowell, 2009; Campbell and Humphries, 2011). Engagement of the cytoplasmic domains of the β subunit causes the receptor to change from a bent or folded non-adhesive conformation to an activated open one (Abram and Lowell, 2009; Campbell and Humphries, 2011). Thus, chemoattractants and chemokines can rapidly regulate integrin receptor clustering, affinity and avidity (Abram and Lowell, 2009; Campbell and Humphries, 2011). Once integrin receptors are in an open state, adhesion-dependent outside-in signaling across the membrane can be triggered by the subsequent binding of ligands to integrin receptors (Figure 6) (Abram and Lowell, 2009). In order to independently assess the influence of inside-out and outside-in signaling to integrin activation, the inside-out step can be bypassed by using an “engineered” multivalent peptide poly-RGD (Arg-Gly-Asp), a ligand that is of sufficient valency to directly aggregate integrins and initiate “outside-in” signaling from all three βfamilies (Ruoslahti, 1996).

In TNFα- and fMLP-primed PMNs, activation of integrins induces oxidative burst after binding of β1 integrin to fibronectin and β2 integrin to fibrinogen (Berton et al., 1992; Mocsai et al., 2002, 2006; Clemens et al., 2004; Gakidis et al., 2004; Jakus et al., 2009; Volmering et al., 2016; Boras et al., 2017). Likewise, crosslinking of the β2 chain by either fibrinogen or specific monoclonal antibodies is sufficient to elicit strong superoxide production in human PMNs, indicating that the β2 integrin family can activate ROS production (Berton et al., 1992). The β2 family includes three well-known members, including LFA-1 (α_L_β_2_; CD11α/CD18) and Mac-1 (also known as CR3, α_m_β2, or CD11b/CD18), which bind to endothelial ICAM-1 (and the complement component, iC3b) and are involved in different phases of PMN transendothelial migration (Abram and Lowell, 2009; Kolaczkowska and Kubes, 2013). Binding of ICAM-1 to the β2 integrin during migration is not sufficient to induce ROS production (Kolaczkowska and Kubes, 2013), demonstrating that other required factors at the site of infection contribute to the regulation of NADPH oxidase. The third member is complement receptor 4 (α_X_β2; CD11c/CD18), which binds to complement factor inactivated-C3b.

β2 integrin ligation leads to the activation of SFKs (Figure 6). PMNs express three different SFKs, Hck, Fgr, and Lyn, which are all important for outside-in signaling (Lowell et al., 1996; Pereira and Lowell, 2003; Giagulli et al., 2006; Lowell, 2011). While Hck and Fgr have overlapping roles in mediating adhesion-mediated activation by integrins, Lyn acts as a negative regulator without affecting inside-out activation (Lowell et al., 1996; Pereira and Lowell, 2003; Giagulli et al., 2006).

β2 integrin-mediated ROS production also requires Syk activation via ITAM-containing DAP12 and FcRγ adaptor proteins (Figure 6) (Mocsai et al., 2002). PMNs isolated from mice lacking both DAP12 and FcRγ have defects in ROS production, indicating the critical and redundant roles of these proteins in promoting oxidative burst (Mocsai et al., 2006; Jakus et al., 2007; Ivashkiv, 2009). Once phosphorylated, DAP12 and FcRγ activate Syk in a similar manner to FcR-induced activation (Mocsai et al., 2002, 2006). Following direct crosslinking of integrin receptors by poly-RGD, *Syk*^−/−^ murine PMNs, as well as human PMNs treated with Syk inhibitors, fail to activate downstream signaling molecules and release superoxide (Mocsai et al., 2002). Furthermore, PMNs expressing Syk proteins with non-functional Src Homology 2 (SH2) domains fail to induce ROS production in response to integrin stimulation (Mocsai et al., 2006). This suggests a mechanism by which Syk interacts with the ITAM domains of DAP12 and FcRγ to propagate signals downstream of integrin receptors. As with FcR signal-transduction pathways, activated Syk recruits and activates Btk and PI3K, which have also been implicated in integrin-mediated generation of oxidative burst (Kulkani et al., 2011; Fumagalli et al., 2013; Volmering et al., 2016). *Btk*^−/−^ PMNs fail to produce superoxide when plated on poly-RGD-coated surfaces, both with or without a secondary stimulus (Volmering et al., 2016). Human PMNs treated with inhibitors to PI3Kγ and PI3Kβ fail to produce ROS when primed with TNFα or fMLP and stimulated with fibrinogen, or when stimulated with RGD (Kulkani et al., 2011; Fumagalli et al., 2013).

As with FcRs, SLP-76, PLCγ2, and Vav are also required for ROS production downstream of integrin stimulation (Myung et al., 2001; Newbrough et al., 2003; Graham et al., 2007; Jakus et al., 2009; Boras et al., 2017). Prior to integrin-mediated ROS production, SLP-76 is required for the phosphorylation of PLCγ2 (Newbrough et al., 2003). The loss of PLCγ2 results in the loss of phosphorylation of Pyk-2, ERK, and, surprisingly, SFK activity following poly-RGD stimulation (Cremasco et al., 2008). Taken together, these data suggest that in addition to its role downstream of SLP-76, PLCγ2 may utilize a feedback loop to further regulate SFKs. Thus, PLCγ2 appears to function both upstream and downstream of SLP-76.

Interestingly, β2 integrin-mediated NADPH oxidase activation also requires Src kinase-associated phosphoprotein 2 (SKAP2) (Boras et al., 2017). SKAP2 is a cytosolic adaptor protein that has been implicated in cell adhesion through its association with integrins and cytoplasmic actin (Togni et al., 2005). SKAP2^−/−^ PMNs fail to produce superoxide when stimulated with RGD and produce significantly less ROS when stimulated with ICAM-1 and fibrinogen in the presence of TNFα (Boras et al., 2017). The loss of SKAP2 results in the loss of phosphorylation of ERK and reduction in the level of Akt phosphorylation (Boras et al., 2017). Specifically, SKAP2 interacts with and activates ADAP, RIAM, and Sirpα in macrophages (Konigsberger et al., 2010; Alenghat et al., 2012) While ADAP is poorly expressed in PMNs, its homolog, PRAM-1, is highly expressed in PMNs (Clemens et al., 2004; Rolan et al., 2013) and is required for ROS production downstream of integrin engagement, as *PRAM-1*^−/−^ PMNs produce lower levels of superoxide following integrin activation (Clemens et al., 2004). Interestingly, PRAM-1 is not required for the activation of other molecules in the SLP-76-dependent pathway (Clemens et al., 2004). However, *PRAM1*^−/−^ PMNs still express low levels of ADAP, so it is possible that in the absence of PRAM1, ADAP plays a redundant role in integrin signal transduction, despite its poor expression.

## Assembly and activation of NADPH oxidase

Priming and activation of the NADPH oxidase leads to translocation of the cytosolic components and phosphorylation of several of these components to ultimate result in a fully assembled and activated complex. Here we provide an overview of these steps. For two recent excellent in-depth reviews of the molecular steps leading to a fully active complex, see Nunes et al. (2013); El-Benna et al. (2016).

### Trafficking of phox units during priming and activation

In resting cells, most of the cytb_558_ are located in the membrane of specific granules, gelatinase-rich granules, and secretory vesicles, rather than the plasma membrane (Borregaard et al., 1983; Jesaitis et al., 1990; Heyworth et al., 1991; Nunes et al., 2013; El-Benna et al., 2016). Priming can induce changes in the subcellular localization of cytb_558_ (Borregaard et al., 1983; Jesaitis et al., 1990; DeLeo et al., 1998; Ward et al., 2000; El-Benna et al., 2016) (Figure 1). In primed cells, levels of cytb_558_ at the plasma membrane increases significantly, which is thought to be due to exocytosis of granules (Borregaard et al., 1983; DeLeo et al., 1998; Ward et al., 2000). In addition, LPS-priming increases the expression of p47^phox^ on the plasma membrane (DeLeo et al., 1998). Changes in subcellular location involve a number of factors. For instance, priming can induce actin cytoskeletal rearrangement and phox proteins have been shown to interact with actin-associated proteins (Sheppard et al., 2005). SNARE proteins, including SNAP-23, play a central role in intracellular membrane trafficking, and inhibition of exocytosis with the fusion protein TAT-SNAP23 reduced plasma membrane expression of gp91^phox^ during priming and ROS production (Uriarte et al., 2011). It is thought that LPS- and TNFα- induced exocytosis is controlled by p38MAPK (Ward et al., 2000), through its regulation of actin cytoskeleton reorganization (McLeish et al., 2017). In addition, inhibition of clathrin-mediated endocytosis prevents TNFα-mediated priming of ROS production by inhibiting granule exocytosis but not p47^phox^ phosphorylation (Creed et al., 2017). Furthermore, murine PMNs defective in Rab27, a small GTPase, exhibit impaired exocytosis, plasma membrane-associated NADPH activity, and overall ROS production following PMA stimulation (Johnson et al., 2010).

Activation by phagocytosis or stimulation with some bacterial products leads to the complete assembly of oxidase components with cytb_558_ and the cytosolic complex residing at the phagosome or plasma membrane (Borregaard et al., 1983; Nunes et al., 2013). The cytosolic complex can be detected as early as 30 seconds after the onset of phagocytosis associated with cytb_558_ (Allen et al., 1999; DeLeo et al., 1999; Karimi et al., 2014). In addition, Rac2 becomes activated via the exchange of guanosine diphosphate (GDP) for guanosine triphosphate (GTP) and translocates to the membrane independently of the p47^phox^/p40^phox^/p67^phox^ complex (Heyworth et al., 1994; Kim and Dinauer, 2006). Activation and assembly of all phox subunits as well as Rac to cytb_558_ is essential for efficient complex function (Heyworth et al., 1991; Leusen et al., 1994a,b; Karimi et al., 2014). Data from biochemical and structural studies show that p47^phox^ and p67^phox^ can bind to two different sites of cytb_558_ independently of each other (Paclet et al., 2000; Maehara et al., 2010; Marcoux et al., 2010). Importantly, the binding of one cytosolic subunit to the cytb_558_ can induce conformational changes leading to increasing affinity of the other cytosolic subunit (Karimi et al., 2014). The tightly bound complex of p47^phox^, p67^phox^, and Rac is critical for the stability of the oxidase in cell-free system (Miyano et al., 2003; Karimi et al., 2014). p47^phox^ and p40^phox^ regulate the assembly and stability of the complex at the plasma and phagosomal membrane, respectively, via the interaction between its PX domain and phosphatidylserine (Matute et al., 2009; Li et al., 2010; Nunes et al., 2013). The Rab27 effector, Munc13-4 is also required for integration of p22^phox^ into the plasma membrane, extracellular ROS production following fMLP stimulation, and intracellular ROS production following infection with serum-opsonized *P. aeruginosa* (Monfregola et al., 2012).

### Phosphorylation of phox subunits

A number of components of the NADPH oxidase complex are phosphorylated during activation. Phosphorylation of phox subunits can directly modulate NADPH oxidase activity and assembly; this has been extensively reviewed (Bokoch et al., 2009; El-Benna et al., 2016). Phosphorylation of p22^phox^ correlates with NADPH oxidase activity, and is mediated by both phospholidase D (PLD)-dependent and -independent pathways (Regier et al., 2000). PLD-independent phosphorylation is phorbol myristate acetate (PMA)-dependent suggesting a role for PKC. Although the importance of phosphorylated p22^phox^ in neutrophils' NADPH oxidase activity has not been studied to our knowledge, work in CHO cells suggested that PMA-dependent phosphorylation of p22^phox^ at threonine 147 is important for its interaction with p47^phox^ (Lewis et al., 2010). Similarly, the PKC-mediated phosphorylation of gp91^phox^ subunit in human PMNs can enhance its binding to Rac2, p67^phox^, and p47^phox^ as well as increase its enzymatic activity (Raad et al., 2009).

The p67^phox^ subunit is constitutively phosphorylated in resting human PMNs and MEK1/2 in a PKC-, PI3K-, and p38MAPK-independent manner although the significance of this is unclear (Dang et al., 2011). Stimulation of cells can further increase p67^phox^ phosphorylation (Dang et al., 2011). Work in cell free systems and murine PMNs also show that p67^phox^ can be phosphorylated by ERK2 and p38MAPK (Dang et al., 2003).

As stated above, p47^phox^ and p40^phox^ are required for the assembly, activation, and stability of the NAPDH oxidase complex at the plasma and phagosomal membranes, respectively (El-Benna et al., 2009; Li et al., 2010; Nunes et al., 2013). It has been shown that Akt, PKC, ERK, and p38 play differing roles in phosphorylating p47^phox^, depending on which surface receptor is activated. It is important to consider that these interactions were determined using experimental models that include cell-free systems, different cell types, as well as in the treatments and techniques used. During active infection or prolonged sterile inflammatory conditions, these proteins may act coordinately to generate high levels of ROS, and may contribute to the dysregulation of NAPDH oxidase during autoimmune or chronic inflammatory diseases.

Structurally, p47^phox^ consists of a phox homology domain (PX), two adjacent SRC homology 3 (SH3) domains, a polybasic autoinhibitory region (AIR), and a proline-rich region (Groemping and Rittinger, 2005; El-Benna et al., 2009). Due to its phosphoinositide-binding ability, specifically for phosphatidylinositol 3,4-biphosphate (PtdIns(3,4)*P*_2_), the p47^phox^ PX domain is primarily responsible for anchoring the p47^phox^/p40^phox^/p67^phox^ complex to the plasma membrane (Zhan et al., 2002; Groemping and Rittinger, 2005; Li et al., 2010) and likely for directing the NADPH oxidase complex to the precise site of the phagocytosed pathogen (Ding et al., 1995; El Benna et al., 1996; El-Benna et al., 2009; Dekker et al., 2000; Dewas et al., 2000; Dang et al., 2001; Chen et al., 2003).

In the resting state, the SH3, and to some extent, PX domains are masked by AIR keeping the protein in an autoinhibited conformation (Ago et al., 1999; Huang and Kleinberg, 1999; Karathanassis et al., 2002; Groemping and Rittinger, 2005). Upon extracellular stimulation, multiple serine-threonine residues within the carboxy-terminal are sequentially phosphorylated leading to conformational changes allowing the SH3 and PX domains to interact with the proline-rich region of the p22^phox^ and PtdIns(3,4)*P*_2_, respectively (Ago et al., 1999; Huang and Kleinberg, 1999; El-Benna et al., 2009; Meijles et al., 2014). Phosphorylation of two serine residues are critical for activating p47^phox^ by inducing conformational changes: Ser345 and Ser379 (Dang et al., 2006; Meijles et al., 2014). As described above, Ser345 is a target during priming of PMNs and for Pin1 binding. While Ser379 is also thought to function as a molecular switch that is important for p47^phox^ conformational changes, the specific kinase and priming agents mediate this phosphorylation is currently unclear (El-Benna et al., 2008; Meijles et al., 2014). However, phosphorylation of these sites relaxes the interaction between AIR and SH3 domains as well as exposes other amino acids to phosphorylation (such as Ser303, 304, 328, 370, and 379) by members of PKC family (Fontayne et al., 2002). Differential phosphorylation of p47^phox^ by PKCα, β, δ, and ζ at different residues leads to the binding of p47^phox^ to p22^phox^, and the activation of NADPH oxidase in a cell-free system (Fontayne et al., 2002). This further exposes the binding pocket for p22^phox^, brings p67^phox^ and p40^phox^ in proximity of cytb_558_, and allows PX domain to bind to PtdIns(3,4)*P*_2_ and phosphatidic acid (Shiose and Sumimoto, 2000; Kanai et al., 2001; Bokoch et al., 2009; El-Benna et al., 2009; Li et al., 2010; Meijles et al., 2014).

Structurally, p40^phox^ consists of a PX, SH3, and PB1 domain; the PB1 domain is important for interacting with p67^phox^ and for an extensive discussion on p40^phox^, see Nunes et al. (2013). *p40*^*phox*−/−^ murine PMNs have reduced p67^phox^ expression and are unable to produce ROS in response to soluble stimuli such as TNFα/GM-CSF-primed fMLP stimulated, serum-opsonized *S. aureus*, and β2 adhesion (Ellson et al., 2006). In addition, p40^phox^ is required for fungal-activated ROS production in human neutrophils (Bagaitkar et al., 2012). Another study in murine PMNs has suggested that PKC-δ-mediated phosphorylation of p40^phox^ at Thr154 is important for IgG particle-stimulated ROS production (Chessa et al., 2010). In contrast to other phox subunits, p40^phox^ plays specialized role in regulating phagocytosis-induced NADPH oxidase via its PX domain. In addition, in some settings, p40^phox^ and p47^phox^ may act cooperatively to recruit the cytosolic complex to the phagosomal membrane (Nunes et al., 2013). A CGD patient expressing a mutated PX domain in the p40^phox^ subunit suffered from granulomatous colitis and his PMNs were unable to produce ROS in response to complement-opsonized *S. aureus* (Matute et al., 2009). Additionally, p40^phox^ has been implicated in the resolution of intestinal inflammation in a DSS-colitis model by regulating *Ccr1* expression in PMNs and expression of enzymes responsible for glycan modifications (Conway et al., 2012). Its PI(3)P binding has also been suggested to control the regulating inflammation in sterile inflammation model by mediating the recruitment of PMNs and macrophages as well as efferocytosis (Bagaitkar et al., 2017).

### Small G proteins

Small G proteins, especially those belonging to the Rho family of small guanine triphosphatase (GTPases), play an important role in the regulation of NADPH oxidase (Miyano and Sumimoto, 2012). Specifically, the Rac proteins, which include Rac1, Rac2, and Rac3, act as important molecular switches in several distinct signaling pathways, including those regulating the actin cytoskeleton. Like other GTPases, these Rac proteins exist in two conformations and the conversion between their inactive and active states is tightly regulated by several protein families, including GEFs, GAPs, and a guanine nucleotide dissociation inhibitor (Rho-GDI) (Hodge and Ridley, 2016). In its resting state, the inactive form of Rac is bound to GDP and is sequestered in the cytosol by its interaction with Rho-GDI (Grizot et al., 2001; Miyano and Sumimoto, 2012; Hodge and Ridley, 2016). Upon receptor stimulation, signaling proteins, such as PIP_3_ and G_βγ_ subunits (Hawkins et al., 2010) activate GEFs to promote the exchange of GDP for GTP, leading to the dissociation of Rho-GDI from Rac and allowing it to translocate to the membrane and interact with downstream effector proteins.

While Rac1 and Rac3 are ubiquitously expressed, Rac2 is expressed only in hematopoietic cells (Grizot et al., 2001; Filippi et al., 2004). Human PMNs primarily express Rac2, while murine PMNs express comparable levels of Rac1 and Rac2. Despite the 98% shared homology between Rac1 and Rac2, studies using genetic knockout mouse models have determined that Rac2 is the critical isoform for NADPH regulation in PMNs (Roberts et al., 1999; Kim and Dinauer, 2001; Gu et al., 2003). *Rac2*^−/−^ PMNs isolated from mice have a defect in superoxide production, where production is restored upon reintroduction of wild-type Rac2 using retrovirus-mediated gene transfer (Filippi et al., 2004). Additionally, PMNs isolated from a patient with a dominant-negative mutation in the gene encoding Rac2 exhibit decreased oxidative activity, underlying the importance of Rac2 in facilitating superoxide burst (Ambruso et al., 1999).

While genetic knockout models have shown that Rac2 is important for NADPH oxidase activity in mice, many biochemical studies have utilized Rac1 to examine the interactions between the Rac GTPase and NADPH oxidase components (Heyworth et al., 1994; Diebold and Bokoch, 2001; Kim and Dinauer, 2001, 2006; Sarfstein et al., 2004; Carstanjen et al., 2005; Maehara et al., 2010). Both Rac isoforms share three highly conserved functional domains, including switch region I, switch region II, and insert region (Lapouge et al., 2000). Thus, it is likely that Rac1 and Rac2 can interact with similar NADPH complex proteins. The primary difference between Rac1 and Rac2 seems to be their locations in the resting cell, which may dictate the upstream signaling proteins that come in contact with Rac2 vs. Rac1 (Tao et al., 2002; Filippi et al., 2004). The functions of Rac2 are dependent on its C-terminal RQQKRP sequence (Tao et al., 2002; Filippi et al., 2004), as well as its ability to translocate from the central cytoplasmic and perinuclear spaces in the cell to the periphery (Filippi et al., 2004; Miyano and Sumimoto, 2012). Rac2 interacts directly with cytb_558_ and is required for the electron transfer reactions mediated by the cytb_558_ complex (Diebold and Bokoch, 2001). Rac2 does not influence the translocation of the p47^phox^/p40^phox^/p67^phox^ complex (Kim and Dinauer, 2006). Rather, Rac1 and Rac2 can directly bind to p67^phox^ via their conserved switch region 1 (Koga et al., 1999; Lapouge et al., 2000; Miyano and Sumimoto, 2012). Based on studies in Rac1, this binding induces conformational changes in p67^phox^ that allow it to bind to gp91^phox^; this interaction is required for oxidative burst (Sarfstein et al., 2004; Maehara et al., 2010). Interestingly, work in a yeast two-hybrid system demonstrated that GTP-bound Rac2 has greater affinity to p67^phox^ than GTP-bound Rac1. However, it is currently unclear whether there are other explanations as to why Rac2 is more important for NADPH oxidase activation or whether its RQQKRP sequence can influence any other p67^phox^-independent downstream pathways. It is worth noting that superoxide production is partially restored in *Rac2*^−/−^ PMNs previously primed with certain stimuli, such as TNF-α or elicited by thioglycollate, suggesting that Rac1 might be sufficient to activate the NADPH oxidase in primed PMNs (Roberts et al., 1999).

At least two other small Rho GTPases, RhoG and Rap1A, can regulate superoxide production. Another member of the Rac subfamily, RhoG, shares 72% amino acid homology with Rac1 (Condliffe et al., 2006) and is important for ROS production under some conditions. However, its role is likely in the transmission of signals from receptors that eventually contribute to the activation of Rac1 and Rac2, rather than any direct involvement in the assembly of the NADPH oxidase itself (Condliffe et al., 2006; Damoulakis et al., 2014). Rap1A, a small GTPase from a different family, is also believed to promote activation of the NADPH oxidase complex. While it is known that Rap1A localizes to the membrane and associates with cytb_558_, its role in NADPH activation is currently poorly defined (Takahashi et al., 2013).

Although activation of NADPH oxidase is short-lived, the coordination of sustaining and then terminating of NADPH oxidase activity at the plasma membrane or during phagosome maturation is not well-characterized (Nunes et al., 2013). However, based on the discussion of the mechanisms of NADPH oxidase activation above, potential mechanisms of deactivation can include the dephosphorylation of phox subunits, activity of GAPs on Rac proteins, and disassembly of the complex. Evidence for and against some of these mechanisms can be found here (Decoursey and Ligeti, 2005).

## Bacterial defenses against ROS

### Intrinsic mechanisms of ROS protection

Bacteria encounter a variety of damaging ROS after activation of the NADPH oxidase complex in PMNs. Upon release, superoxide anion (${\text{O}}_{2}^{-}$), the byproduct of the electron transport chain, undergoes spontaneous or enzymatic dismutation to hydrogen peroxide (H_2_O_2_). H_2_O_2_ can then oxidize ferrous iron to generate highly reactive hydroxyl radical OH· through a mechanism known as the Fenton reaction. Additionally, upon oxidative burst in PMNs the granule-localized enzyme myeloperoxidase (MPO) converts hydrogen peroxide into the highly bactericidal hypochlorous acid (HOCl) at neutral or low pH, which is believed to enhance clearance of pathogens (Figure 2) (Klebanoff, 1970; Rosen and Klebanoff, 1979; Foote et al., 1983; Klebanoff et al., 2013; Levine and Segal, 2016). These oxygen derivatives have the capacity to restrict bacterial growth during tissue infections, as they can diffuse through the membranes of both intracellular and extracellular bacterial pathogens and damage their DNA, protein, and lipid molecules. Bacteria, in turn, have developed a number of strategies to resist killing by ROS, including detoxification of these radical species into less damaging byproducts, as well as through the repair of damaged molecular and cellular targets. Many of these strategies likely evolved as mechanisms for bacteria to adapt to the entrance of oxygen into the earth's atmosphere nearly 2.4 billion years ago (Fischer et al., 2016), and were later adapted and altered by pathogens to respond to ROS encountered in host environments. Such activities can be classified as “intrinsic” resistance mechanisms. Additionally, several bacterial pathogens employ “extrinsic” resistance mechanisms to directly suppress ROS production by interfering with the activity of the NADPH oxidase complex.

#### Detoxification and scavenging of ROS

A number of bacterial enzymes, including superoxide dismutases (SODs), catalases, and peroxiredoxins, are utilized to transform ROS into less toxic products (Imlay, 2008). Catalases and peroxiredoxins function as H_2_O_2_ scavengers. Examples of these proteins in *E. coli* include the peroxiredoxin AhpC and the catalase KatG (Imlay, 2008). While these scavengers exhibit some functional redundancy, they typically contribute to detoxification at different H_2_O_2_ concentrations. At low H_2_O_2_ concentrations, AhpC serves as the primary scavenger, whereas KatG becomes the primary scavenging enzyme at high concentrations (Hillar et al., 2000; Seaver and Imlay, 2001). In *E. coli* and many organisms, the transcriptional response to H_2_O_2_ is dependent on the global regulator OxyR (Imlay, 2008). Oxidation of this protein typically occurs when micromolar concentrations of H_2_O_2_ are encountered, typically as a result of exposure to exogenous sources of oxidative stress (Altuvia et al., 1997; Aslund et al., 1999). Once oxidized, OxyR undergoes a conformational change that allows for the binding of a large number of DNA promoter sequences (Zheng et al., 1998). The OxyR regulon includes genes encoding peroxiredoxins and catalases, as well as several other factors important for responding to oxidative damage, including the reducing agents glutathione reductase (*gor*), glutaredoxin 1 (*grxA)*, and thioredoxin 2 (*trxC*), which function to minimize the frequency of aberrant disulfide bond formation occurring as a result of exposure to ROS (Zheng et al., 1998, 2001; Imlay, 2008).

In contrast to the peroxiredoxins and catalases, which scavenge H_2_O_2_, SODs scavenge superoxide. *E. coli* encodes two cytoplasmic SOD isozymes, one, MnSOD (SodA), which uses the co-factor manganese, and another, FeSOD, (SodB), which uses the co-factor iron (Imlay, 2008). Additionally, because superoxide does not easily cross membranes at a neutral pH, *E. coli* also secretes another, copper- and zinc-co-factored SOD, CuZnSOD (SodC) into the periplasm (Korshunov and Imlay, 2002). Interestingly, SODs may also reduce overall H_2_O_2_ levels by preventing further interaction of superoxide with other reductants in the cell (Liochev and Fridovich, 1994). While baseline expression of SODs is usually high (Imlay and Fridovich, 1991), activation of the SoxRS regulatory system further enhances expression of these enzymes in response to superoxide stress (Liochev et al., 1999). Much like OxyR, SoxR undergoes a conformational change upon detection of redox stress (Hassan and Fridovich, 1977). In this case, SoxR contains an iron sulfur cluster, which, upon oxidation, induces a structural change in that protein; oxidized SoxR then promotes increased transcription of the DNA-binding protein SoxS (Hidalgo et al., 1997). In *E. coli*, SoxS positively regulates about a dozen genes, including those encoding SODs and several other genes involved in detoxification, iron-sulfur cluster repair, and drug efflux (Imlay, 2008). Some species of bacteria, such as *Pseudomonas aeruginosa*, lack a SoxS homolog, and instead encode a SoxR protein that serves as both the redox sensor and direct inducer of the regulon (Kobayashi and Tagawa, 2004; Eiamphungporn et al., 2006).

#### Iron sequestration

Because free iron is susceptible to Fenton chemistry, bacteria utilize a number of mechanisms to sequester iron or control its uptake in response to encountering ROS in the environment (Liochev and Fridovich, 1994; Keyer and Imlay, 1996; Imlay, 2006, 2008). In gram-negative bacteria, iron homeostasis is primarily controlled by the transcriptional regulatory protein Fur, which becomes activated upon binding of ferrous iron (Bagg and Neilands, 1987). Additionally, the transcription of Fur is promoted by OxyR and SoxR, underlying the importance of iron regulation in the face of oxidative attack (Zheng et al., 1999). Activated Fur represses the transport of iron in the bacterial cell and, during periods of oxidative stress, may function to minimize the availability of ferrous iron (Troxell and Hassan, 2013). Curiously, in *Salmonella typhimurium*, activation of Fur could have an inhibitory effect on its ability to “extrinsically” resist ROS, as Fur negatively regulates the expression of the SPI2 pathogenicity island (Choi et al., 2014). As will be discussed below, the SPI2 pathogenicity island is important for suppression of oxidative burst by *S. typhimurium* (Vazquez-Torres and Fang, 2001; Vazquez-Torres et al., 2001); however, the interplay between Fur activation and NADPH oxidase inhibition by this pathogen has not been fully delineated. Additionally, a family of proteins known as ferritins acts to sequester iron and maintain iron homeostasis. In particular, the ferritin-like protein Dps is critical for withstanding oxidative stress, as it both sequesters iron and binds DNA to protect it from damage (Halsey et al., 2004; Velayudhan et al., 2007). Furthermore, because oxygen radicals can release iron from iron-sulfur clusters, factors that promote regeneration of iron-sulfur clusters can also play a role in recovering from oxidative damage. In *E. coli*, the Suf iron-cluster repair machinery, in particular, plays an important role in recovery from H_2_O_2_-mediated damage (Imlay, 2008; Jang and Imlay, 2010).

#### DNA damage repair

DNA damage is a key consequence of ROS *in vitro* and was believed to be the major mechanism of bacterial killing by ROS, particularly at the concentrations encountered in mammalian tissues (Buchmeier et al., 1995). Oxidation of DNA bases by OH· can produce several damaging byproducts, the most abundant being 8-hydroxyguanine, which frequently mismatches with adenine. Additionally, ribose oxidation by ROS can induce strand breaks in bacterial DNA (Imlay, 2008). Bacteria encode numerous DNA repair enzymes that are crucial for recovering from ROS attack (Imlay, 2008). Many of these factors are essential for growth and/or survival under aerobic conditions. However, the expression of some of these proteins is enhanced under oxidative stress conditions in an OxyR and/or SoxRS-dependent manner, suggesting that increased levels of some DNA repair factors are required for resistance to ROS (Zheng et al., 2001; Imlay, 2008). Chief among these factors are the proteins involved in the base excision repair (BER) pathway, which scan DNA for the absence of duplex integrity and strand breaks and facilitate repairs. These include endonuclease IV (Nfo) and exonuclease III (Xth) (Demple et al., 1983; Van Sluys et al., 1986). Additionally, DNA damage by oxidative stress frequently activates the SOS response pathway, which induces the UvrABC excision nuclease as well as the Rec recombinational machinery (Imlay and Linn, 1987).

#### Challenges of modeling intrinsic bacterial defenses against PMN-derived ROS

It is important to note that the chemistry of oxidants in the neutrophil phagosome is far different from that of the cell's surroundings or that of dilute solutions to which oxidants have been added. Additionally, ROS in the context of immune cells and tissues play pleiotropic roles in bacterial clearance by acting to trigger other anti-bactericidal activities of the host. Thus, phenotypes observed in patients and animals could arise from the failure of events dependent on ROS production but are not directly due to ROS themselves. Following oxidative burst, bacteria are in close contact with the phagosomal membrane and therefore there is little space between the bacterium and the source of oxidative burst (Winterbourn et al., 2016). Additionally, oxidants are produced with tightly regulated kinetics and may react with host cell components as well as with bacterial targets (Winterbourn et al., 2006). Furthermore, the vacuole pH of PMNs has been shown to be basic (Levine and Segal, 2016). Because the peroxidatic and chlorinating activities of MPO require an acidic environment, it is unclear whether MPO catalyzes the formation of HOCl within completely closed phagosomes (Levine and Segal, 2016). Thus, while much work has dissected the behavior of bacterial mutants in cultures containing exogenous oxidants, it is challenging to use these *in vitro* models to predict the relative contribution of bacterial factors to resisting oxidative species produced by NADPH oxidase during PMN encounters.

Furthermore, studies examining the sensitivity of various bacterial species to oxidative killing by neutrophils suggest that these organisms are not uniformly sensitive to the oxidative burst of these cells. For example, the pathogen *S. typhimurium* is no more susceptible to PMN-mediated killing when it is unable to mount a transcriptional response to ROS (Papp-Szabo et al., 1994). Furthermore, studies performed with *E. coli*, the primary organism used to model bacterial mechanisms of ROS resistance, have found that this bacterium is rapidly killed by PMNs through non-oxidative mechanisms (Rada et al., 2004). Additionally, the pathogen *S. pneumoniae* is cleared by PMNs even when NADPH oxidase is inhibited (Standish and Weiser, 2009). However, some bacterial pathogens are readily killed by the oxidative burst of PMNs. The pathogen *S. aureus* is killed by PMNs through an NADPH oxidase-dependent mechanism (Rada et al., 2004). Accordingly, infections with *S. aureus* are commonly associated with CGD (Buvelot et al., 2017).

In some cases, it can be challenging to dissect the role of ROS production in bacterial killing by PMNs, as PMNs employ several bactericidal functions that act downstream of NADPH oxidase activation, including the release of NETs and activation of certain proteases (Reeves et al., 2002; Rada et al., 2004; Fuchs et al., 2007). Further, ROS themselves can interact with other host-derived factors to exacerbate bacterial killing. For example, ${\text{O}}_{2}^{-}$ can react with NO· to generate the highly toxic peroxynitrite (OONO^−^) anion (Brunelli et al., 1995). Moreover, it is challenging to model bacterial-PMN interactions *in vitro*, as conditions such as multiplicity of infection, and expression of virulence and stress response factors can be manipulated to mask the bactericidal effects of PMN-derived ROS.

Nonetheless, several studies have demonstrated a role for ROS-detoxifying and/or repair enzymes during mammalian bacterial infection models, suggesting that the role of these factors in protecting against host-derived ROS must not be fully discounted (De Groote et al., 1997; Roggenkamp et al., 1997; Harris et al., 2003; Brenot et al., 2004; Hebrard et al., 2009; Aussel et al., 2011; Green et al., 2016; Honn et al., 2017). Therefore, although *in vitro* models do not fully replicate the oxidative environment bacterial pathogens encounter in the host, the concepts outlined above provide us with a framework to begin to understand the mechanisms by which bacterial pathogens detoxify mammalian sources of ROS or other host factors triggered by ROS and repair damage to cellular targets.

### Extrinsic mechanisms of ROS resistance: bacterial suppression of oxidative burst

**Figure 7:**
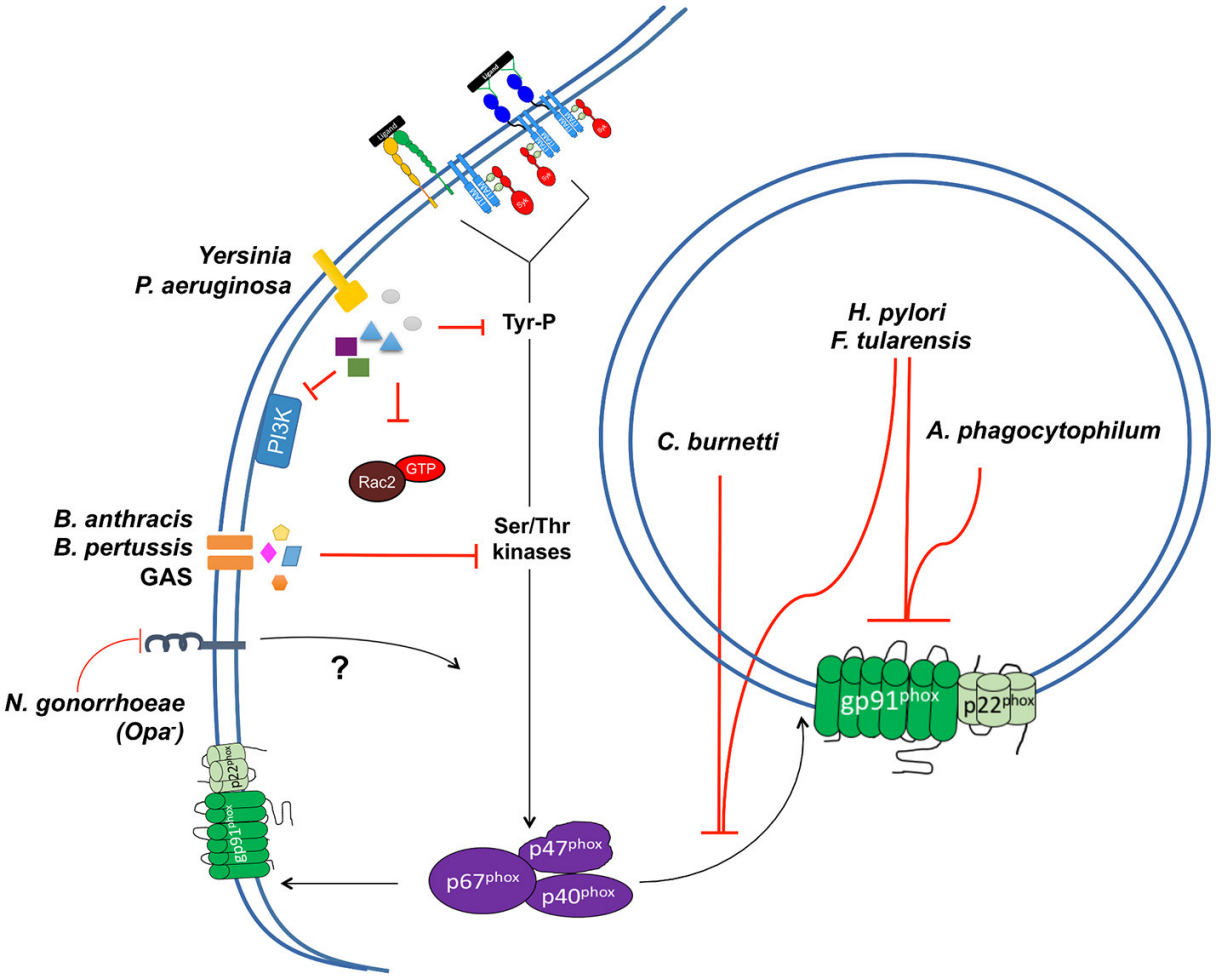
Mechanisms of NADPH oxidase inhibition by bacterial pathogens in PMNs. Several bacterial pathogens employ mechanisms to interfere with the activation and/or localization of the NADPH complex of PMNs. These include strategies to prevent oxidative burst in the phagosomal compartment. Three pathogens, *F. tularensis, A. phagocytophilum*, and *H. pylori*, exclude one or both components of the cytb_558_ complex from the phagosomal membrane. Three pathogens, *F. tularensis H. pylori*, and *C. burnetti*, exclude or prevent p67^phox^/p40^phox^/p47^phox^ from binding to the phagosomal membrane. A number of extracellular pathogens also employ mechanisms to inhibit oxidative burst. *P. aeruginosa* inhibits the oxidative burst of PMNs through the activities of two T3SS effectors, ExoS and ExoT. Both effectors inhibit activation of PI3K signaling pathways upstream of p67^phox^/p40^phox^/p47^phox^ activation. The pathogenic *Yersinia* sp. inhibit respiratory burst in PMNs, though their activities have been largely modeled in other phagocytic cell types. *Y. pseudotuberuclosis* translocates the effector protein YopE through a T3SS to block activation of Rac in HL-60 cells. *Y. pseudotuberculosis* also translocates another T3SS effector protein, YopH, which interferes with oxidative burst in macrophages. The effects of YopH on oxidative burst have not been examined in PMNs; it dismantles the SLP-76/SKAP2 signal transduction pathway in these cells, suggesting that interference of this pathway in PMNs could prevent oxidative burst. Three pathogens, *B. anthracis, B. pertussis*, and Group A *Streptococcus* (GAS), also secrete toxins into PMNs that interfere with signaling pathways required for oxidative burst. Finally, strains of *N. gonorrhoeae* lacking opacity-associated proteins do not activate oxidative burst in PMNs, though the mechanism by which this occurs remains unclear. It is hypothesized that the failure of opacity-negative strains to engage CEACAM receptors could result in a failure to stimulate kinase signaling upstream of p47^phox^ activation. Alternatively, it is possible that opacity-negative strains may actively block trafficking of NADPH oxidase components to membrane sites. Additionally, three other pathogens, *L. monocytogenes, S. typhimurium*, and *V. parahaemolyticus*, are capable of inhibiting the oxidative burst in cultured cells; however, their effects on neutrophils have not been examined in detail.

Bacterial pathogens also employ a number of measures to prevent exposure to increased levels of ROS at sites of infection. One method by which pathogens prevent exposure to ROS is through suppression of or interference with oxidative burst in phagocytes, particularly PMNs, usually through the actions of secreted effector proteins or toxins (Figure 7). This can occur through a number of mechanisms, the studies of which have provided valuable insights into the mechanisms of NADPH oxidase activation in PMNs and other phagocytic cells.

Several pathogens capable of multiplying within the phagosomal compartment of PMNs or other phagocytes secrete proteins or toxins that prevent the activated NADPH oxidase complex from assembling at the phagosomal membrane, effectively blocking oxidative burst at this location (Figure 7). For example, while *Helicobacter pylori* induces a rapid oxidative burst in cultured PMNs, this burst is limited to the extracellular space. Biochemical and microscopic examination of *H. pylori-*infected PMNs found that the phagosomes of these cells acquire cytb_558_, but at decreased levels compared to phagosomes of PMNs treated with other stimulants. Consequently, *H. pylori-*infected phagosomes are unable to recruit or retain the p47^phox^ and p67^phox^ subunits (Allen et al., 2005). Interestingly, this phenomenon is dependent on phagocytosis of unopsonized bacteria, as treatment of *H. pylori* with serum prior to PMN infection induces a modest respiratory burst that is contained within the phagosomal compartment (Allen et al., 2005). The mechanism by which *H. pylori* prevents trafficking of NADPH oxidase to the phagosome is not known, however it is hypothesized to be linked to alterations in granule targeting, as *H. pylori-*infected phagosomes also lack the granule-specific marker lactoferrin (Allen et al., 2005).

Similarly, microscopic studies of PMN infections by the obligate intracellular pathogen *Anaplasma phagocytophilum* have shown that this bacterium prevents the assembly of cytb_558_ at the phagosomal membrane. Curiously, PMNs infected with both *E. coli* and *A. phagocytophilum* recruit cytb_558_ to *E. coli-*containing phagosomes but not to the membranes of phagosomes containing *A. phagocytophilum*. This finding indicates that *A. phagocytophilum* does not suppress a global respiratory burst, and may instead selectively exclude cytb_558_ from the phagosomal membrane (IJdo and Mueller, 2004). The obligate intracellular pathogen *Coxiella burnetti* also prevents respiratory burst in PMNs following phagocytosis (Siemsen et al., 2009). This inhibition is believed to be localized to the bacteria-containing phagosome, as treatment of *C. burnetti*-infected PMNs with a soluble agonist does not prevent a respiratory burst in response to the agonist (Siemsen et al., 2009). Membrane fractions isolated from PMNs infected with *C. burnetti* do not contain p47^phox^ and p67^phox^ subunits, suggesting that recruitment of these subunits to the phagosome may be inhibited by this pathogen (Siemsen et al., 2009). The mechanism by which this occurs is not known, though it is hypothesized that the secreted acid phosphatase ACP may contribute to this phenotype. Indeed, treatment of PMNs with recombinant ACP blocks oxidative burst in response to PMA; however the contribution of this protein to *C. burnetti* infection of PMNs has not been determined (Hill and Samuel, 2011).

*S. typhimurium* uses factors encoded within the SPI-2 pathogencity island to exclude cytb_558_ from the phagosomal membrane of macrophages (Vazquez-Torres et al., 2000; Gallois et al., 2001). Consequently, these phagosomes do not recruit p47^phox^ and p67^phox^ (Gallois et al., 2001). While the precise mechanism for this phenotype is not known, it is hypothesized to be mediated by one or more type 3 secretion system (T3SS) effectors that may alter proximal signaling events upstream of cytb_558_ recruitment to the phagosome (Gallois et al., 2001). However, this phenotype appears to be limited to *S. typhimurium*-infected macrophages, as PMNs restrict *S. typhimurium* growth in an NADPH oxidase-dependent manner during murine infection (Burton et al., 2014).

The intracellular pathogen *Listeria monocytogenes* also prevents NADPH oxidase from assembling at the phagosomal membrane of macrophages, through a mechanism attributed to secretion of the pore forming toxin listeriolysin O (Lam et al., 2011). However, this phenotype has only been observed in macrophages, and is not apparent in PMNs where *L. monocytogenes* induces ROS production, and is rapidly killed by these cells (Kobayashi et al., 2003).

A number of pathogens inhibit respiratory burst in PMNs or other phagocytes by directly targeting the signaling pathways that lead to activation and assembly of the NADPH oxidase complex (Figure 7). Pathogenic species of the genus *Yersinia* utilize two T3SS effectors, YopH and YopE, to suppress the oxidative burst of macrophages and HL-60 cells, respectively (Bliska and Black, 1995; Songsungthong et al., 2010). YopE, a GAP (GTPase activating protein) inhibits oxidative burst in the PMN-like HL-60 cell line after fMLP stimulation and YopH prevents Fc-receptor mediated oxidative burst in macrophages, through an unknown mechanism (Bliska and Black, 1995; Songsungthong et al., 2010). While no published studies have examined the contributions of these two effectors to inhibition of oxidative burst in PMNs, given that *Yersinia pestis* represses oxidative burst in human PMNs in a T3SS-dependent manner, it is likely that one or both of these effectors prevent ROS production in PMNs (Spinner et al., 2008). Notably, YopH, a powerful phosphotyrosine phosphatase, targets the PRAM-1/SKAP-HOM and the SLP-76/Vav/PLCγ2 signal transduction axes of PMNs during tissue infection (Rolan et al., 2013). While this work did not examine the effects of YopH on oxidative burst in these cells, it is possible that interference of this pathway by YopH leads to suppression of oxidative burst in PMNs.

The intracellular pathogen *Fransicella tularensis* suppresses oxidative burst in the phagosome of PMNs as well as blocking the oxidative burst of PMNs treated with a number of heterologous stimuli. This is believed to occur through a multifaceted strategy that involves excluding gp91^phox^ from the phagosomal membrane, diminishing p47^phox^ phosphorylation, and inhibiting NADPH oxidase activity through a post-assembly mechanism (McCaffrey and Allen, 2006). Genetic studies have attributed this phenotype the global transcriptional regulator FevR (McCaffrey et al., 2010).

*P. aeruginosa* utilizes two T3SS-translocated effectors, ExoS and ExoT, to block oxidative burst in human PMNs (Vareechon et al., 2017). Secretion of one or both of these effectors inhibits PI3K signaling upstream of p47^phox^ and p40^phox^ phosphorylation and is dependent their ADP-ribosyltransferase activities. ExoS ribosylates Ras, which prevents it from interacting with and activating PI3K (Vareechon et al., 2017). While ExoT has been shown to interfere with PI3K signaling as well, its target remains unclear (Vareechon et al., 2017).

*Vibrio parahaemolyticus* utilizes two T3SS effectors, VopS and VopL, to inhibit oxidative burst in COS cells transfected with the NADPH oxidase components. Inhibition by VopS is believed to be a result of AMPylation of the RhoGTPase Rac by this effector, effectively blocking it from interacting with other members of the NADPH oxidase complex (Woolery et al., 2014). By contrast, VopL disrupts the normal assembly of the actin cytoskeleton of host cells, thereby preventing translocation of p47^phox^, p67^phox^ and Rac to the membrane (de Souza Santos et al., 2017). However, the effects of VopL and VopS on oxidative burst in PMNs have not yet been examined.

A number of toxins secreted by bacterial pathogens are able to block oxidative burst in PMNs (Figure 7). These include the lethal and edema toxins of *Bacillus anthracis*, the streptolysin O toxin of Group A *Streptococcus* (GAS) and the CyaA toxin of *Bordatella* pertussis. Lethal toxin proteolytically cleaves mitogen-activated protein kinase kinases, which are involved in MAP kinase signaling upstream of p47^phox^ activation (Crawford et al., 2006). Edema toxin and CyaA both block oxidative burst in PMNs by catalyzing the unregulated conversion of cytosolic ATP to cAMP (Crawford et al., 2006; Cerny et al., 2017). Enhanced levels of cAMP, in turn, inhibit oxidative burst through two converging mechanisms. The first involves the aberrant activation of SHP-1, resulting in reduced activation of MAP kinase signaling upstream of p47^phox^ phosphorylation, and the second involves the activation of Epac (the exchange protein directly activated by cAMP), which promotes inhibition of PLC through an unknown mechanism (Cerny et al., 2017). Streptolysin O also blocks oxidative burst in PMNs infected with GAS, as well as in PMNs stimulated with PMA, suggesting that this toxin may interfere with one or more signaling pathways upstream of oxidative burst (Uchiyama et al., 2015).

Finally, at least one pathogen, *Neisseria gonorrhoeae*, may regulate expression of its outer membrane components to prevent activation of NADPH oxidase. While *N. gonorrhoeae* can stimulate oxidative burst in PMNs (Simons et al., 2005), this activation is diminished following infection of PMNs with strains lacking pili or opacity-associated proteins (Fischer and Rest, 1988; Smirnov et al., 2014). Although the mechanism for this phenotype is currently unknown, it is hypothesized to be a consequence of the failure of these strains to engage the carcinoembryonic antigen-related cell adhesion molecule (CEACAM) receptors, which stimulate kinase signaling cascades upstream of p47^phox^ activation (Criss and Seifert, 2008; Smirnov et al., 2014). Alternatively, it is possible that opacity-negative mutants may actively block trafficking of cytb_558_ to sites of *N. gonorrhoeae* uptake. Thus, it remains unclear whether *N. gonorrhoeae* prevents oxidative burst in PMNs by evading activation of signaling pathways upstream of oxidative burst, or if it directly inhibits signaling required for its activation.

### Resisting PMN-derived ROS: a balance between extrinsic and intrinsic defenses?

Interestingly, while suppression of oxidative burst by some pathogens is advantageous for mammalian infections, many of these organisms, including *S. typhimurium, Yersinia* sp, GAS, *F. tularensis*, and *H. pylori*, also require mechanisms to detoxify ROS or repair their damage in animal models of infection (De Groote et al., 1997; Roggenkamp et al., 1997; Harris et al., 2003; Brenot et al., 2004; Hebrard et al., 2009; Green et al., 2016; Honn et al., 2017). These data suggest that suppression of NADPH oxidase activity by secreted effector proteins and toxins is not sufficient to defend these bacterial pathogens against oxidative attack and that these factors must work in concert with cellular processes critical for detoxification and repair to prevent restriction by host-derived ROS. These findings may reflect the fact that much of the work studying inhibition of NADPH oxidase by bacterial pathogens has been performed in cell culture models, which typically do not reflect the influence that tissue architecture and bacterial tropisms may have on pathogen survival. Additionally, when modeling bacterial phenotypes in cell culture, pathogens can be manipulated such that they are maximally expressing virulence factors that may not be uniformly expressed in all mammalian tissue sites (Davis et al., 2015). In the case of *S. typhimurium* and *H. pylori*, which can replicate extracellularly in host tissues, ROS detoxification and repair mechanisms may be required to resist killing by extracellular superoxide bursts, which are not inhibited by these pathogens (Vazquez-Torres et al., 2000; Allen et al., 2005). Additionally, *S. typhimurium* that escapes the phagosomal compartment has been shown to experience higher levels of oxidative stress in the cytosol, where bacteria may encounter other sources of ROS (van der Heijden et al., 2015). Similarly, though the extracellular pathogens GAS and *Yersinia* do inhibit extracellular oxidative bursts (Bliska and Black, 1995; Songsungthong et al., 2010; Uchiyama et al., 2015), they may require mechanisms to resist killing by ROS produced by the extracellular oxidative bursts of nearby phagocytic cells that have not been intoxicated with effector proteins and/or toxins from those pathogens. Altogether, these findings underlie the importance of studying mechanisms of bacterial detoxification of ROS and repair of oxidative damage, even in pathogens that inhibit respiratory burst in tissue culture models.

## Conclusions

Understanding how PMNs are activated and how they can become dysregulated will help to develop strategies to maintain the crucial balance between their beneficial and detrimental effects. The tug-of-war between PMN activation and functions and bacterial resistance mechanisms is critical for determining the outcome of the infection. While ROS themselves may both directly kill some organisms while functioning to regulate other bactericidal functions of PMNs, their importance in controlling many pathogens is critical. Unraveling the direct vs. indirect killing mechanisms of ROS will be greatly facilitated by recent technical advances and our increased understanding of the variety of roles PMNs play in host physiology.

## Author contributions

GN, EG, and JM reviewed literature, wrote, and edited the manuscript. GN and EG designed the figures.

### Conflict of interest statement

The authors declare that the research was conducted in the absence of any commercial or financial relationships that could be construed as a potential conflict of interest.
